# OSMAC-Driven Discovery of Six New Alkaloids from the Cold-Seep-Derived Fungus *Talaromyces amestolkiae* HDN21-0307

**DOI:** 10.3390/md23090337

**Published:** 2025-08-25

**Authors:** Xinsheng Huang, Jiajin Wu, Luning Zhou, Zhengjie Wang, Qian Che, Liangzhen Chen, Wenxue Wang, Tianjiao Zhu, Dehai Li

**Affiliations:** 1 Key Laboratory of Marine Drugs Ministry of Education, School of Medicine and Pharmacy, Ocean University of China, Qingdao 266003, China; lepei2019@163.com (X.H.); wjj981123@163.com (J.W.); 18895692529@163.com (L.Z.); 18075005915@163.com (Z.W.); cheqian064@ouc.edu.cn (Q.C.); 2 Qingdao Institute of Innovation, East China University of Science and Technology, Qingdao 266003, China; jean_chenchen@163.com; 3 Laboratory for Marine Drugs and Bioproducts & Laboratory for Marine Biology and Biotechnology, Qingdao Marine Science and Technology Center, Qingdao 266237, China; 4 Sanya Oceanographic Institute, Ocean University of China, Sanya 572025, China

**Keywords:** OSMAC, *Talaromyces amestolkiae*, alkaloids, cyanated derivatives, azaphilone analogue

## Abstract

Six new alkaloid compounds, including two rare aromatic nitrile compounds talaronitriles A–B (**1**–**2**), a novel oxime-functionalized azadiphilone analogue talarooxime A (**3**), a new phenylhydrazone alkaloid talarohydrazone E (**4**), and two new dipeptide compounds talarodipeptides A–B (**5**–**6**), were isolated from the deep-sea cold-seep-derived fungus *Talaromyces amestolkiae* HDN21-0307 via OSMAC approach. Compound **1** is the first natural naphthalene compound with cyano groups. Compound **3** represents the first natural product containing an oxime-functionalized azadiphilone scaffold. Their structures and absolute configurations were elucidated through spectroscopic data analysis and quantum chemical calculations. Notably, compound **3** demonstrated moderate DPPH free-radical-scavenging activity, with an IC_50_ value of 29.41 μM.

## 1. Introduction

Natural alkaloids are nitrogen-containing alkaline substances found in living organisms and represent one of the most significant classes of secondary metabolites produced by marine fungi [1]. These compounds typically possess structurally complex nitrogenous heterocycles. Owing to their unique chemical scaffolds and diverse biological activities—such as antitumor, antibacterial, antiviral, and anti-inflammatory effects—natural alkaloids have garnered increasing interest in drug research and natural product chemistry [1,2].

*Talaromyces* fungi are widely distributed across various environments, including marine habitats, soil, and plants. Species within this genus produce an extensive array of secondary metabolites, among which alkaloids represent a major class. To date, numerous alkaloids have been isolated from *Talaromyces* fungi and can be roughly classified into amide alkaloids, indole alkaloids, terpene alkaloids, and quinoline alkaloids according to their structural types [3,4,5]. Notably, many alkaloids from *Talaromyces*, such as chaetominine B [3] and ditalaromylectones A–B [4], possess not only novel structures but also promising biological activities, underscoring the remarkable metabolic capacity of *Talaromyces* fungi for producing novel alkaloids.

In our previous study, the fungal strain *Talaromyces amestolkiae* HDN21-0307 was isolated from deep-sea cold seep sediments in the South China Sea, yielding four novel phenylhydrazone alkaloids (talarohydrazones A–D) with notable antimicrobial and cytotoxic activities [6]. However, similar to most marine microorganisms, *T. amestolkiae* HDN21-0307 still harbors numerous biosynthetic gene clusters that remain silent under monoculture conditions, limiting the discovery of novel bioactive metabolites [7]. The OSMAC (One Strain Many Compounds) strategy, which involves altering medium composition and cultivation conditions, has been widely employed to activate these silent gene clusters [8]. To date, this approach remains the most convenient and efficient strategy for mining microbial metabolites [9].

Consequently, the OSMAC strategy was also applied to further explore the metabolites of *T. amestolkiae* HDN21-0307. Six new alkaloids (**1**–**6**) were isolated from *T. amestolkiae* HDN21-0307 cultivated under two distinct conditions: (1) shaking conditions in Modified Glucose Minerals Salts 2# liquid medium and (2) static conditions in rice solid media with the addition of NaBr. Notably, these included the first reported naphthalene-fused nitrile natural product (**1**) and the inaugural oxime-functionalized azaphenanthrenone alkaloid (**3**). Additionally, compound **3** showed moderate DPPH free-radical-scavenging activity, with an IC_50_ value of 29.41 μM. Herein, details of the isolation, structure elucidation, and bioactivity evaluation of these compounds are presented.

## 2. Results

**Figure 1:**
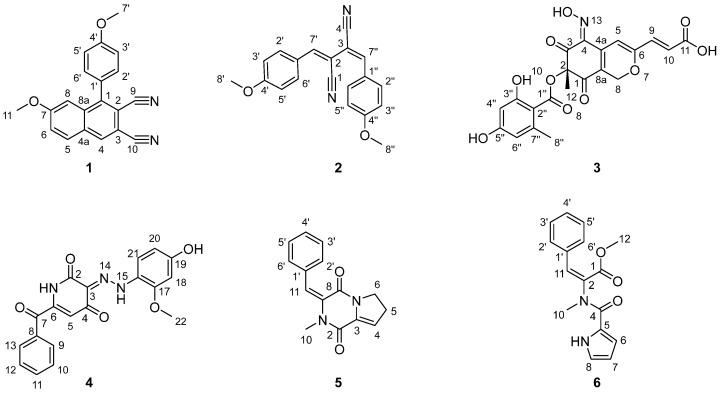
Structures of the isolated compounds **1**–**6**.

The OSMAC strategy was applied to comprehensively explore the metabolic potential of *T. amestolkiae* HDN21-0307 by employing six different culture media under shaking or static cultivation conditions. As a result, this strain exhibited distinct UV absorption profiles compared with those of previously known compounds from *T. amestolkiae* HDN21-0307 [6] under the following conditions: (1) modified Glucose Mineral Salts 2# liquid medium under shaking conditions and (2) rice solid medium supplemented with NaBr under static conditions (Figure S1). To elucidate the structures corresponding to these characteristic peaks, large-scale fermentations were conducted under both conditions, followed by ethyl acetate and methanol extraction. The resulting extracts were subjected to extensive column chromatography over silica gel, ODS, and Sephadex LH-20, ultimately yielding six new alkaloids (**1**–**6**) (Figure 1).

Compound (**1**), obtained as a colorless powder, has a molecular formula of C_20_H_14_N_2_O_2_ based on HRESIMS (*m*/*z* 315.1128, [M + H]^+^ calcd. for C_20_H_15_N_2_O_2_ 315.1124), indicating fifteen degrees of unsaturation. Analysis of 1D NMR and HSQC (Table 1) indicated the existence of eighteen aromatic carbons, including ten non-hydrogenated carbons (*δ*_C_ 108.5, 111.7, 117.0, 117.7, 128.9, 130.5, 136.5, 148.2, 162.2, and 162.5), and two oxymethyl carbons (*δ*_C_ 55.9 and 56.0). The characteristic hydrogen signals H-2′/H-6′ (*δ*_H_ 7.42, d, *J* = 8.7 Hz) and H-3′/H-5′ (*δ*_H_ 7.18, d, *J* = 8.7 Hz) indicated the presence of a para-substituted benzene ring. Among them, the substituent at the 4′-position of the benzene ring was unambiguously identified as a methoxy group (C-7′), as confirmed by its characteristic chemical shift (C-4′, *δ*_C_ 162.2; C-7′, *δ*_C_ 55.9) and key HMBC correlation from H-7′ (*δ*_H_ 3.93, s) to C-4′. Additionally, a trisubstituted benzene ring with C-7 oxygen methyl group was determined through characteristic signals of H-5 (*δ*_H_ 8.07, d, *J* = 9.0 Hz), H-6 (*δ*_H_ 7.46, dd, *J* = 9.0, 2.5 Hz), and H-8 (*δ*_H_ 7.06, d, *J* = 2.5 Hz) and the HMBC correlations from H-11 (*δ*_H_ 3.77, s) to C-7 (*δ*_C_ 162.9), from H-5 to C-7/C-8a (*δ*_C_ 163.0), from H-6 to C-4a (*δ*_C_ 130.5), and from H-8 to C-4a/C-6 (*δ*_C_ 123.8)/C-7.

The established structural fragments account for eight degrees of unsaturation, leaving seven remaining unsaturation to be assigned. Analysis of the NMR data indicated only six aromatic carbons (*δ*_C_ 108.5, 111.7, 117.0, 117.7, 136.2, and 148.2) and two nitrogen atoms remained unassigned. To satisfy the high degree of unsaturation, compound **1** likely contained an additional aromatic ring. The key HMBC correlations of H-8/C-1, H-5/C-4 (*δ*_C_ 163.2), and H-4/C-2/C-5/C-8a confirmed the pentasubstituted aromatic ring as part of a poly-substituted naphthalene scaffold. Furthermore, the remaining two non-protonated aromatic carbons (C-9, *δ*_C_ 117.0; C-10, *δ*_C_ 117.7) and two nitrogen atoms needed to account for four additional degrees of unsaturation, leading to the identification of two cyano groups in compound **1**. Finally, the para-substituted benzene ring was determined to be connected to the C-1 of the naphthalene scaffold through the correlations between H-2′/H-6′ and C-1 (*δ*_C_ 148.2), while the remaining two cyanide groups were connected to C-2 (*δ*_C_ 111.7) and C-3 (*δ*_C_ 108.5), respectively. In addition, the presence of a cyano group was also confirmed by its characteristic infrared absorption band at 2227 cm^−1^. The structure of **1** was conclusively established and named talaronitrile A [10].

The cyano group is widely used in the pharmaceutical industry for its ability to enhance binding affinity, improve pharmacokinetic properties, and reduce drug resistance [11,12]. Naturally occurring nitriles, mostly existing as cyanogenic glycosides, are widely distributed across various organisms including plants, fungi, and marine species [11,12]. Aromatic nitrile-containing natural products are relatively rare [11,12]. Notably, compound **1** represents the first natural product featuring a naphthalene-linked nitrile scaffold.

**Figure 2:**
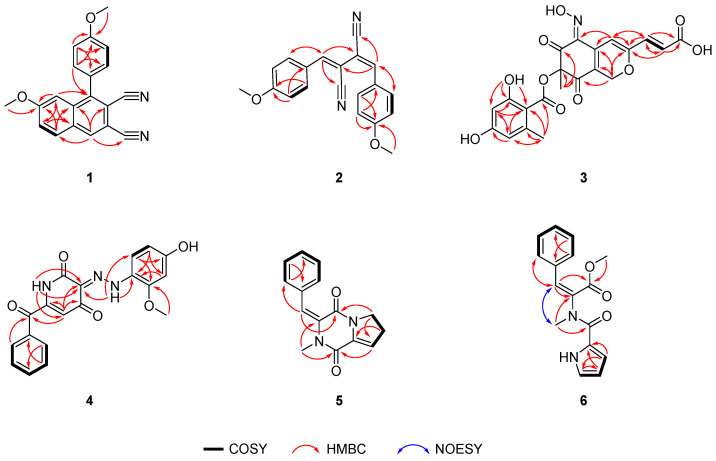
Key COSY, key HMBC, and key NOESY correlations of compounds **1**–**6**.

Compound (**2**) was obtained as a pale-yellow powder with a molecular formula of C_20_H_16_N_2_O_2_, based on HRESIMS data (*m*/*z* 317.1294 [M + H]^+^ calcd. for C_20_H_17_N_2_O_2_, 317.1285). Comparative analysis of the 1D NMR and HSQC spectra (Table 1) revealed the existence of sixteen aromatic carbons, including eight non-hydrogenated carbons (*δ*_C_ 100.8, 106.1, 116.1, 119.1, 125.4, 125.4, 161.9, and 162.7) and two oxymethyl carbons (*δ*_C_ 55.6 and 55.7). Compound **2** and epurpurin C shared the similar nitrile skeleton, as evidenced by the HMBC correlations from H-7′ (*δ*_H_ 7.35, s) to C-1 (*δ*_C_ 119.1)/C-3/C-2′, from H-2′/6′ (*δ*_H_ 7.41, d, *J* = 8.9 Hz) to C-1′/C-4′ (*δ*_C_ 161.9)/C-7′, from H-3′/5′ (*δ*_H_ 6.93, d, *J* = 8.9 Hz) to C-1′/C-4′, from H-7″ (*δ*_H_ 7.46, s) to C-2/C-4 (*δ*_C_ 116.1)/C-2″, from H-2″/H-6″ (*δ*_H_ 7.87, d, *J* = 8.9 Hz) to C-1″/C-4″ (*δ*_C_ 162.7)/C-7″, and from H-3″/5″ (*δ*_H_ 6.98, d, *J* = 8.9 Hz) to C-1″/C-4″ (Figure 2) [13]. The only difference between **2** and epurpurin C was that the absolute configuration of Δ^3,7″^ in **2** was *E*, supported by the existence of asymmetrical peaks in 1D NMR. Additionally, the C-H coupling constants (^3^*J*_C-4,H-7″_ = 14.6 Hz) further corroborated the double-bond configurations of Δ^3,7″^ (Figure S18) [14]. Hence, the structure of **2** was established and named talaronitrile B.

Compound (**3**), obtained as a bright yellow powder, has a molecular formula of C_21_H_17_NO_10_, based on HRESIMS data (*m*/*z* 444.0927 [M + H]^+^ calcd. for C_21_H_18_NO_10_, 444.0925). The 1D NMR and HSQC spectra (Table 2) revealed the presence of seventeen aromatic carbons, including thirteen non-hydrogenated carbons (*δ*_C_ 86.9, 104.3, 120.0, 141.3, 142.4, 145.0, 158.8, 164.4, 166.6, 166.9, 170.5, 185.4, and 189.0), five methine carbons (*δ*_C_ 101.8, 104.9, 113.0, 124.4, and 137.3), one methylene carbon (*δ*_C_ 64.6), and two methyl carbons (*δ*_C_ 21.9, 24.3). The ^3^*J*_H-9,H-10_ (15.5 Hz) unambiguously assigned the *E* configuration of the double bond Δ^9,10^. Comparative NMR data revealed that compound **3** and talarohydrazone B share an essentially identical core structure (Figure 2). The key structural differences include the absence of a phenylhydrazone moiety in compound **3**, along with the presence of an additional nitrogen atom and hydroxyl group. The remaining degree of unsaturation, combined with the characteristic downfield shift of C-4 (*δ*_C_ 142.4), suggested the presence of an oxime moiety at C-4 in **3**. Consequently, the planar structure of **3** was established and designated as talarooxime A.

**Figure 3:**
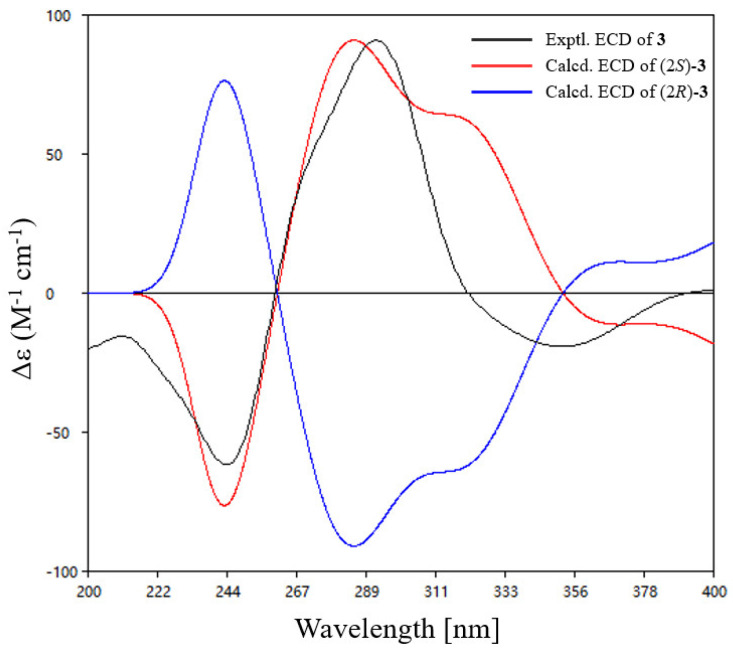
Experimental and calculated ECD spectra of compound **3**.

Due to the lack of diagnostic NOESY correlations, the configuration of the Δ^4,N−13^ double bond could not be determined spectroscopically. To resolve this, the time-dependent density-functional theory (TDDFT) calculations were performed on both possible diastereomers 4*E*-**3** (**3a**) and 4*Z*-**3** (**3b**) (Figure S55). DP4+ probability analysis demonstrated excellent agreement (99.94%) between **3b** and the experimental NMR data, establishing the *Z*-configuration at Δ^4,N−13^ double bond (Figure S56). The C-2 configuration was assigned as consistent with talarohydrazone B based on biosynthetic considerations. Furthermore, (2*S*)-**3** showed remarkable concordance between experimental and calculated ECD spectra (Figure 3), thereby confirming the absolute configuration at C-2. Thus, the absolute configuration of compound **3** was determined to be (2*S*, 4*Z*, 9*E*). Structural diversity among currently known azaphilones is predominantly concentrated on chain extension at the C-2/C-6 positions of the compound **3** core scaffold, with modifications at the C-4 position being rare. Furthermore, the substituents introduced are predominantly aliphatic chains or aryl/heteroaryl groups [15]. In contrast, compound **3** represents the first reported oxime-containing azaphilone derivative featuring a substitution at the C-4 position.

Compound (**4**) was isolated as a red amorphous powder. The molecular formula of **4** was determined to be C_19_H_15_N_3_O_5_ based on the HRESIMS date (*m*/*z* 364.0937 [M − H]^−^ calcd. for C_19_H_14_N_3_O_5_, 364.0939), indicating fourteen degrees of unsaturation. Spectroscopic analysis of the ^1^H NMR data of **4** showed signals for three active hydrogen protons (*δ*_H_ 10.30, 11.15, and 17.30), nine olefinic protons, and one methoxy group. Analysis of the ^13^C NMR and HSQC spectra (Table 2) indicated three carbonyl carbons (*δ*_C_ 161.5, 178.9, and 180.5), fifteen olefinic carbons, including six non-hydrogenated carbons, and one oxymethyl (*δ*_C_ 56.1) carbon. The aforementioned information accounted for eleven out of the fourteen degrees of unsaturation, suggesting that **4** was a tricyclic compound. The NMR data of **4** was highly similar to that of talarohydrazone A (Figure 2) [6], with the only difference being the presence of a carbonyl group at C-7 in **4** instead of a methylene group at C-7 in talarohydrazone A. The HMBC correlations from H-5/H-9/H-13 to C-7 (*δ*_C_ 180.5) confirmed this difference between them. Thus, the planar structure of **4** was determined, named talarohydrazone E. The configuration of the double bond Δ^3,14^ could not be determined due to the absence of NOESY correlations. TDDFT was used to calculate NMR data of the two possible configurations 3*E*-**4** (**4a**) and 3*Z*-**4** (**4b**). The improved DP4+ probability analysis showed that **4a** was in agreement with the NMR experimental data with a probability of 100% (Figure S58 and Table S3), confirming the configuration of the double bond Δ^3,14^ as *E* configuration.

Compound (**5**) was purified as a pale-yellow oil with the molecular formula as C_15_H_14_N_2_O_2_ based on the HRESIMS data (*m*/*z* 255.1126, [M + H]^+^ calcd. for C_15_H_15_N_2_O_2_, 255.1128). The ^13^C NMR and HSQC spectra of **5** indicated the presence of fifteen carbon atoms (Table 3), including two carbonyl carbons (*δ*_C_ 156.79 and 156.84,), ten olefinic carbons, two methylenes (*δ*_C_ 28.3 and 46.0,), and one methyl (*δ*_C_ 36.3). The NMR data of **5** closely resembled that of cyclo Pro-1-*N*-methyl-9,11-en-Phe [16], indicating that they possessed the same cyclic dipeptide skeleton. The only structural difference between them lies in the bond between C-3 and C-4: a double bond in **5** versus a single bond in *cyclo Pro-1-N-methyl-9*,*11-en-Phe*. Based on the COSY correlations of H-4 (*δ*_H_ 6.26)/H-5, and the HMBC correlations from H-5 to C-3 (*δ*_C_ 133.5) and from H-6 to C-4 (*δ*_C_ 120.3), the double bond Δ^3^ in **5** was established (Figure 2). Thus, the planar structure of **5** could be determined, named talarodipeptide A. Due to the absence of NOESY correlations of H-10/H-11, the configuration of the double bond Δ^9,11^ was determined by HSQMBC (Figure S44). The C-8 to H-11 (*J*_C,H_ = 9.6 Hz) obtained from the HSQMBC spectrum unambiguously assigned the *E* configuration of the double bond Δ^9,11^ [14].

Compound (**6**) was isolated as a pale-yellow oil. The molecular formula, C_16_H_16_N_2_O_3_, was deduced from its HRESIMS data (*m*/*z* 285.1237, [M + H]^+^ calcd. for C_16_H_17_N_2_O_3_, 285.1234), indicating 10 degrees of unsaturation. The ^13^C NMR and HSQC spectra of **6** indicated (Table 3) two carbonyl carbons (*δ*_C_ 164.8 and 167.1), twelve olefinic carbons, including three non-hydrogenated carbons, and two methyls (*δ*_C_ 35.9 and 53.2,). The NMR data of **6** closely resembled that of Pyrrole-*Phe*-OMe ester [17], indicating that they had the same skeleton. The difference was that the 3-NH of **6** was replaced by a methyl group (C-3, *δ*_C_ 35.9), and C-2 (*δ*_C_ 134.1) and C-11 (*δ*_C_ 137.9) were double-bonded rather than single-bonded, confirmed by the HMBC correlations from H-10 (*δ*_H_ 3.06, s) to C-2/C-4, and from H-2′ to C-11 (Figure 2). The NOESY correlation of H-10/H-11 indicated the *E*-configuration of the double bond Δ^2,11^. Thus, the structure of **6** was determined, named talarodipeptide B.

Antibacterial, antitumor, and the free-radical-scavenging activity tests were conducted on compounds **1**–**6**. Only compound **3** exhibited strong activity (IC_50_ = 29.41 μM) in the free-radical scavenging. Vitamin C (IC_50_ = 14.8 μM) was used as the positive control.

## 3. Materials and Methods

### 3.1. General Experimental Procedures

Ultraviolet (UV) spectroscopy was recorded on a Hitachi 5430 spectrophotometer (Hitachi, Tokyo, Japan). Circular dichroism (CD) spectra and optical rotations were measured by JASCO P-1020 digital polarimeter (JASCO Corporation, Tokyo, Japan), respectively. Infrared (IR) spectra were obtained on a Bruker Tensor-27 spectrophotometer (Bruker, Saarbruecken, Germany) with KBr pellets. High-resolution electrospray ionization mass spectrometry (HRESIMS) data were acquired on a Thermo Scientific LTQ Orbitrap XL mass spectrometer (Thermo Fisher Scientific, Waltham, MA, USA). Nuclear magnetic resonance (NMR) spectra were recorded on a JEOL JNM-ECP 600 MHz spectrometer (JEOL, Beijing, China) and an Agilent 500 MHz DD2 spectrometer (Agilent, Beijing, China), with tetramethylsilane (TMS) as an internal standard. Column chromatography was performed using Sephadex LH-20 (Amersham Biosciences, Uppsala, Sweden) and silica gel (Qingdao Marine Chemical Factory, Qingdao, China). Preparative high-performance liquid chromatography (HPLC) was carried out on a YMC-Pack ODS-A column (10 × 250 mm, 5 μm, 3 mL/min; YMC Co., Ltd., Kyoto, Japan).

### 3.2. Fungal Material and Fermentation

*T*. *amestolkiae* HDN21-0307 (GenBank No. OQ954837) was isolated from deep-sea cold seep sediments in the South China Sea (119°17′005.887″ E, 22°07′02.589″ N). This strain is a facultative anaerobe. Its mycelium, initially off-white, developed a green pigmentation after 4–5 days of incubation. Additionally, the strain produced a diffusible red pigment over time. This strain has been deposited in the Marine Medicinal Bioresources Center, School of Medicine and Pharmacy, Ocean University of China, Qingdao, China.

### 3.3. OSMAC Research, Fermentation, and Extraction

Based on the OSMAC strategy, *T*. *amestolkiae* HDN21-0307 was cultured in two different ways—shaking condition and static condition.

Shaking conditions: Using five distinct liquid media formulations: Modified Fungus 2# liquid media (Table S1. N1), PDB liquid media (Table S1. N2), SDA liquid media (Table S1. N3), Modified Glucose Minerals Salts 1# liquid media (Table S1. N4), and Modified Glucose Minerals Salts 2# liquid media (Table S1. N5). The products of *T*. *amestolkiae* HDN21-0307 cultured in MGMS (Table S1. N5) liquid media 2# under shaking conditions displayed unusual UV absorptions, which was the reason for selecting as the optimal conditions for large-scale fermentation. *T*. *amestolkiae* HDN21-0307 was cultured in 500 mL Erlenmeyer flasks, containing 150 mL of GMS liquid media for 9 days under shaking conditions (180 rpm, 28 °C, pH 7.0) in seawater (collected from JiaoZhou Bay, Qingdao, China). A total of 45 L of broth was extracted using EtOAc (3 × 45 L) to obtain crude extract (45.0 g).

Static conditions: Using two distinct solid media formulations: Rice solid media (Table S1. N6), NaBr rice solid media (Table S1. N7). The HPLC analysis of extracts revealed the chromatogram of the extract from the strain HDN21-0307 cultured in rice media with the addition of NaBr exhibited different peaks compared to rice media with no addition. Therefore, rice media with the addition of NaBr was selected as the optimal condition for large-scale fermentation. The fungus was cultured in 500 mL Erlenmeyer flasks each containing 80 g rice, 1 g NaBr and 120 mL freshwater at 28 °C for 30 days. A total of 12 L of broth was extracted using methanol (3 × 12 L) to obtain crude extract (100.0 g).

### 3.4. Separation and Purification of Compounds

The shaking crude extract was fractionated into five subcomponents (Fr.A-Fr.E) through VLC column chromatography, utilizing a stepped gradient elution protocol with MeOH-CH_2_Cl_2_. Fr.C was separated by ODS column to obtain five subfractions (Fr.C.1-Fr.C.5). Fr. C.2 was purified by Sephadex LH-20 and semi-preparative HPLC (YMC-pack ODS, 10 × 250 mm, 3.0 mL/min) to afford **3** (3.2 mg, *t_R_* = 25 min). Fr.C.4 was purified by semi-preparative HPLC to afford **1** (3.2 mg, *t_R_* = 31 min). Fr.C.4 was purified by semi-preparative HPLC to afford **2** (2.1 mg, *t_R_* = 29 min).

The static crude extract also used the same steps to separate the extract into the five subfractions (Fr.A-E). Fr. C was separated by ODS column to obtain four subfractions (Fr.C.1-Fr.C.4). Fr. C.1 was purified by semi-preparative HPLC to afford **5** (5.0 mg, *t*_R_ = 24 min). Fr.C.2 was purified by semi-preparative HPLC to afford **6** (6.0 mg, *t*_R_ = 24 min). Fr.C.3 was purified by semi-preparative HPLC to afford **4** (2.0 mg, *t*_R_ = 25 min).

Talaronitrile A (**1**): white powder, UV (MeOH) *λ*_max_ 223 (0.3), 258 (1.0) nm; IR (KBr) *ν*_max_ 2925, 2227, 1677, 1614, 1206 cm^−1^; ^1^H and ^13^C NMR data, Table 1; HRESIMS *m*/*z* 315.1124 [M + H]^+^ (calcd. for C_20_H_15_N_2_O_2_, 315.1128).

Talaronitrile B (**2**): faint yellow powder, UV (MeOH) *λ*_max_ 241 (0.3), 311 (0.4) nm, 360 (0.5) nm; IR (KBr) *ν*_max_ 3419, 2921, 2221, 1602, 1510, 1263, 1178, cm^−1^; ^1^H and ^13^C NMR data, Table 1; HRESIMS *m*/*z* 317.1294 [M + H]^+^ (calcd. for C_20_H_17_N_2_O_2_, 317.1285).

Talarooxime A (**3**): green yellow powder, ${\left[\alpha \right]}_{D}^{25}$ 150 (MeOH); UV (MeOH) *λ*_max_ 215 (1.0), 265 (1.0) nm, 406 (0.4) nm; IR (KBr) *ν*_max_ 1646, 1536, 1324, 1267, 1172 cm-1; ^1^H and ^13^C NMR data, Table 2; HRESIMS *m*/*z* 444.0927 [M + H]^+^ (calcd. for C_21_H_18_NO_10_, 444.0925).

Talarohydrazone E (**4**): red amorphous powder; UV (MeOH) *λ*_max_ 206 (1.86), 258 (0.81), 510 (1.39) nm; IR (KBr) *ν*_max_ 3446, 2923, 1626, 1417, 1307, 1284, 1024 cm^−1^; ^1^H and ^13^C NMR data, Table 2; HRESIMS *m*/*z* 364.0937 [M − H]^−^ (calcd. for C_19_H_14_N_3_O_5_, 364.0939).

Talarodipeptide A (**5**): pale-yellow oil; UV (MeOH) *λ*_max_ 225 (0.52), 308 (1.25) nm; IR (KBr) *ν*_max_ 3436, 1682, 1435, 1136, 760 cm^−1^; ^1^H and ^13^C NMR data, Table 3; HRESIMS *m*/*z* 255.1126 [M + H]^+^ (calcd. for C_15_H_15_N_2_O_2_, 255.1128).

Talarodipeptide B (**6**): pale-yellow oil; UV (MeOH) *λ*_max_ 220 (0.65), 274 (1.02) nm; IR (KBr) *ν*_max_ 3279, 1717, 1612, 1544, 1437, 762 cm^−1^; ^1^H and ^13^C NMR data, Table 3; HRESIMS *m*/*z* 285.1237 [M + H]^+^ (calcd. for C_16_H_17_N_2_O_3_, 285.1234).

### 3.5. Computation Section

The details of ECD calculation for **3** and the NMR calculation for **3**–**4** are available in the Supporting Information (Figures S54–S58 and Tables S2 and S3).

### 3.6. Assay of Activity

DPPH activity. The free-radical clearance experiment is based on the Sharma method [18] and has been modified. Compound **1**–**3** and vitamin C are dissolved in anhydrous ethanol and diluted into 6 gradients. Mix the vortex of these samples evenly, avoid light at room temperature for 30 min, and read the absorption at 515 nm as a positive control.

The ability to scavenge the DPPH was calculated according to the equation:

DPPH free-radical-scavenging rate D_VC_% = [(A_blank_ − A_postive control_) ÷ A_blank_] × 100%.

DPPH free-radical-scavenging rate D_sample_% = [[A_blank_ − (A_sample_ − A_control_)] ÷ A_blank_] × 100%.

A_blank_: the absorbance of the DPPH solution;

A_postive control_: the absorbance of the solution with VC;

A_control_: absorbance of the sample.

Antibacterial activity. Antibacterial activities of compounds **1**–**6** were evaluated against *Bacillus cereus* ATCC 4342, MRCNS, MRSA, *Staphylococcus aureus* ATCC 29213, *Klebsiella pneumoniae* ATCC 43816, *Escherichia coli* ATCC 25922, *Enterococcus faecalis* ATCC 29212, *Pseudomonas aeruginosa* ATCC 27853, and *Bacillus subtilis* ATCC 6051. Ciprofloxacin was used as a positive control. The detailed methodologies for biological testing have been described in a previous report [19].

Cytotoxicity Assay. Cytotoxic activities of compounds **1**, **4**–**6** were evaluated against K562 (using the MTT method), MDA-MB-231, NCI-H446, NCI-H446/EP, and ASPC-1 (using the SRB method) cell lines. Adriamycin was used as a positive control. The detailed methodologies for biological testing have been described in previous reports [20,21].

## 4. Conclusions

In summary, the application of the OSMAC strategy successfully activated the secondary metabolic potential of *T. amestolkiae* HDN21-0307, leading to the isolation of six new alkaloids. Among these, compounds (**1**) and (**2**), isolated for the first time from the genus Talaromyces, represent cyano-containing natural products. Notably, compound (**1**) constitutes the first naturally occurring cyano-substituted naphthalene derivative, while compound (**3**) is the first natural product featuring an azaphilone scaffold bearing an oxime functional group. This result emphasizes the significance of the OSMAC approach in unlocking the metabolic potential of microorganisms and further demonstrates the substantial potential for discovery within the secondary metabolites of fungi originating from extreme habitats such as cold springs.

## Figures and Tables

**Table 1:** ^1^H NMR (400 MHz) and ^13^C NMR (125 MHz) data of **1** in methanol-*d*_4_ and **2** in chloroform-*d* (*δ* in ppm, *J* in Hz).

No.	1		No.	2	
	*δ*_C_, Type	*δ*_H_ (*J* in Hz)		*δ*_C_, Type	*δ*_H_ (*J* in Hz)
1	148.2, C		1	119.1, C	
2	111.7, C		2	106.1, C	
3	108.5, C		3	100.8, C	
4	136.2, CH	8.50, s	4	116.1, C	
4a	130.5, C		1′	125.4, C	
5	132.2, C	8.07, d (9.0)	2′	132.5, CH	7.41, d (8.9)
6	123.8, CH	7.46, dd (9.0, 2.5)	3′	114.4, CH	6.93, d (8.9)
7	162.9, CH		4′	161.9, C	
8	107.1, CH	7.06, d (2.5)	5′	114.4, CH	6.93, d (8.9)
8a	136.5, C		6′	132.5, CH	7.41, d (8.9)
9	117.0, C		7′	146.1, CH	7.35, s
10	117.7, C		8′	55.6, CH_3_	3.88, s
11	56.0, CH_3_	3.77, s	1″	125.4, C	
1′	128.9, C		2″	132.1, CH	7.87, d (8.9)
2′	132.3, CH	7.42, d (8.7)	3″	114.8, CH	6.98, d (8.9)
3′	115.5, CH	7.18, d (8.7)	4″	162.7, C	
4′	162.2, C		5″	114.8, CH	6.98, d (8.9)
5′	115.5, CH	7.18, d (8.7)	6″	132.1, CH	7.87, d (8.9)
6′	132.3, CH	7.42, d (8.7)	7″	149.0, CH	7.46, s
7′	55.9, CH_3_	3.93, s	8″	55.7, CH_3_	3.85, s

**Table 2:** ^1^H NMR (500 MHz) and ^13^C NMR (125 MHz) data of **3** in acetone-*d*_6_ and **4** in DMSO-*d*_6_ (*δ* in ppm, *J* in Hz).

No.	3		No.	4	
	*δ*_C_, Type	*δ*_H_ (*J* in Hz)		*δ*_C_, Type	*δ*_H_ (*J* in Hz)
1	189.0, C		1		
2	86.9, C		2	160.9, C	
3	185.4, C		3	127.9, C	
4	142.4, C		4	178.2, C	
4a	120.0, C		5	108.6, CH	5.91, s
5	104.9, CH	6.56, s	6	145.1, C	
6	158.8, C		7	180.5, C	
8	64.6, CH_2_	5.14, m	8	134.6, C	
8a	141.3, C		9	129.9, CH	7.91, m ^a^
9	137.3, CH	7.23, d (15.5)	10	129.0, CH	7.61, m ^a^
10	124.4, CH	6.42, d (15.5)	11	134.4, CH	7.76, m ^a^
11	166.9, CH		12	128.6, CH	7.61, m ^a^
12	21.9, CH_3_	1.77, s	13	129.1, CH	7.91, m ^a^
1″	170.5, C		16	122.6, C	
2″	104.3, C		17	151.6, C	
3″	166.6, C		18	99.7, CH	6.62, d (2.3)
4″	101.8, CH	6.25, d (2.4)	19	159.8, C	
5″	164.4, C		20	109.3, CH	6.57, dd (8.8, 2.1)
6″	113.0, CH	6.38, d (2.1)	21	117.9, CH	7.69, d (8.3)
7″	145.0, C		22	56.3, CH_3_	3.92, s
8″	24.3, CH_3_	2.62, s	1	-NH	11.15, s
3″	-OH	10.68, s	15	-NH	17.30, s
			19	-OH	10.30, s

^a^ Signals were overlapped.

**Table 3:** ^1^H NMR (400 MHz) and ^13^C NMR (100 MHz) data of **5** in chloroform-*d* and **6** in methanol-*d*_4_ (*δ* in ppm, *J* in Hz).

No.	5		No.	6	
	*δ*_C_, Type	*δ*_H_ (*J* in Hz)		*δ*_C_, Type	*δ*_H_ (*J* in Hz)
1			1	167.1, C	
2	156.8, C		2	134.1, C	
3	133.5, C		3		
4	120.3, CH	6.26, t (3.1)	4	164.8, C	
5	28.3, CH_2_	2.84, td (9.1, 3.1)	5	126.1, C	
6	46.0, CH_2_	4.13, t (9.1)	6	114.7, CH	6.53, dd (3.8, 1.1)
7			7	110.5, CH	6.03, dd (3.8, 2.6)
8	156.79, C		8	123.6, CH	6.87, dd (2.4, 1.2)
9	131.7, C		9		
10	56.3, CH_3_	2.91, s	10	35.9, CH_3_	3.06, s
11	119.6, CH	7.30, s	11	137.9, CH	7.63, s
12			12	53.2, CH_3_	3.68, s
1′	134.7, C		1′	134.0, C	
2′	129.4, CH	7.22, t (7.5)	2′	131.2, CH	7.61, m^a^
3′	128.3, CH	7.33, m^a^	3′	130.2, CH	7.40, m ^a^
4′	128.2, CH	7.33, m^a^	4′	131.9, CH	7.40, m ^a^
5′	128.3, CH	7.33, m^a^	5′	130.2, CH	7.40, m ^a^
6′	128.3, CH	7.22, t (7.5)	6′	131.2, CH	7.61, m ^a^

^a^ Signals were overlapped.

## Data Availability

The original data presented in the study are included in the article/Supplementary Material; further inquiries can be directed to the corresponding author.

## Supplementary Materials

The following supporting information can be downloaded at: https://www.mdpi.com/article/10.3390/md23090337/s1. Figure S1: OSMAC cultivation strategy for *T. amestolkiae* HDN21-0307; Table S1: OSMAC consists of media for *T. amestolkiae* HDN21-0307; Figure S2: The 16S rRNA sequences data of *T. amestolkiae* HDN21-0307; Figure S3: HRESIMS spectrum of compound **1**; Figure S4: ^1^H NMR spectrum (400 MHz, CD_3_OD) of compound **1**; Figure S5: ^13^C NMR spectrum (125 MHz, CD_3_OD) of compound **1**; Figure S6: HSQC spectrum of compound **1**; Figure S7: HMBC spectrum of compound **1**; Figure S8: COSY spectrum of compound **1**; Figure S9: IR spectrum of compound **1**; Figure S10: HRESIMS spectrum of compound **2**; Figure S11: ^1^H NMR spectrum (400 MHz, CDCl_3_) of compound **2**; Figure S12: ^13^C NMR spectrum (125 MHz, CDCl_3_) of compound **2**; Figure S13: HSQC spectrum of compound **2**; Figure S14: HMBC spectrum of compound **2**; Figure S15: COSY spectrum of compound **2**; Figure S16: NOESY spectrum of compound **2**; Figure S17: HSQMBC spectrum of compound **2**; Figure S18: ^3^*J*_C-H_ values obtained from the HSQMBC spectrum (500 MHz, CDCl_3_) of **2**; Figure S19: IR spectrum of compound **2**; Figure S20: HRESIMS spectrum of compound **3**; Figure S21: ^1^H NMR spectrum (500 MHz, Acetone-*d*_6_) of compound **3**; Figure S22: ^13^C NMR spectrum (125 MHz, Acetone-*d*_6_) of compound **3**; Figure S23: HSQC spectrum of compound **3**; Figure S24: HMBC spectrum of compound **3**; Figure S25: COSY spectrum of compound **3**; Figure S26: NOESY spectrum of compound **3**; Figure S27: IR spectrum of compound **3**; Figure S28: HRESIMS spectrum of compound **4**; Figure S29: ^1^H NMR spectrum (500 MHz, DMSO-*d*_6_) of compound **4**; Figure S30: ^13^C NMR spectrum (125 MHz, DMSO-*d*_6_) of compound **4**; Figure S31: HSQC spectrum of compound **4**; Figure S32: HMBC spectrum of compound **4**; Figure S33: COSY spectrum of compound **4**; Figure S34: NOESY spectrum of compound **4**; Figure S35: IR spectrum of compound **4**; Figure S36: HRESIMS spectrum of compound **5**; Figure S37: ^1^H NMR spectrum (400 MHz, CDCl_3_) of compound **5**; Figure S38: ^13^C NMR spectrum (100 MHz, CDCl_3_) of compound **5**; Figure S39: HSQC spectrum of compound **5**; Figure S40: HMBC spectrum of compound **5**; Figure S41: COSY spectrum of compound **5**; Figure S42: NOESY spectrum of compound **5**; Figure S43: HSQMBC spectrum of compound **5**; Figure S44: ^3^*J*_C-H_ values obtained from the HSQMBC spectrum (400 MHz, CDCl_3_) of **5**. Figure S45: IR spectrum of compound **5**; Figure S46: HRESIMS spectrum of compound **6**; Figure S47: ^1^H NMR spectrum (100 MHz, CD_3_OD) of compound **6**; Figure S48: ^13^C NMR spectrum (100 MHz, CD_3_OD) of compound **6**; Figure S49: HSQC spectrum of compound 6; Figure S50: HMBC spectrum of compound **6**; Figure S51: COSY spectrum of compound **6**; Figure S52: NOESY spectrum of compound **6**; Figure S53: IR spectrum of compound **6**; Figure S54: ECD: B3LYP/6-31G(d) optimized lowest energy conformers for **3** [22,23]; Figure S55: B3LYP/6-31G(d) optimized lowest energy conformers for **3**; Figure S56: NMR calculations with DP4+ probability analysis for compounds **3**; Table S2. DP4+ evaluation of theoretical and experimental one of **3** [24]; Figure S57: B3LYP/6-31G(d) optimized lowest energy conformers for **4**; Figure S58: NMR calculations with DP4+ probability analysis for compounds **4**; Table S3. DP4+ evaluation of theoretical and experimental one of **4**.
