# Supplementary material for: OSMAC-Driven Discovery of Six New Alkaloids from the Cold-Seep-Derived Fungus Talaromyces amestolkiae HDN21-0307
Source: Mar Drugs. 2025 Aug 25;23(9):337. doi: 10.3390/md23090337 (PMC12472177; doi:10.3390/md23090337)
Supplement: Supplementary file 1 [file marinedrugs-23-00337-s001.zip › marinedrugs-3826990-supplementary.pdf]

OSMAC-Driven Discovery of Six New Alkaloids from the Cold Seep-Derived Fungus *Talaromyces amestolkiae* HDN21-0307

Xinsheng Huang[1, †], Jiajin Wu[1, †], Luning Zhou[1], Zhengjie Wang[1], Qian Che[1], Liangzhen Chen[2], Wenxue Wang[1, *], Tianjiao Zhu[1, 3, 4, *], and Dehai Li[1, 3, 4, *]

[1] School of Medicine and Pharmacy, Ocean University of China, Qingdao 266003, People's Republic of China

[2] Qingdao Institute of Innovation, East China University of Science and Technology, Qingdao 266003, People's Republic of China

[3] Laboratory for Marine Drugs and Bioproducts of Qingdao National Laboratory for Marine Science and Technology, Qingdao, 266237, People's Republic of China

[4] Sanya Oceanographic Institute, Ocean University of China, Qingdao 266003/Sanya 572025, People's Republic of China;

[†] These authors contributed equally to this work.

*Email: dehaili@ouc.edu.cn, zhutj@ouc.edu.cn, wangwenxue@ouc.edu.cn.

List of Supporting Information

Fig S1: OSMAC cultivation strategy for *T. amestolkiae* HDN21-0307;...... 5

Table S1: OSMAC consists of media for *T. amestolkiae* HDN21-0307; 5

Fig S2: The 16S rRNA sequences data of *T. amestolkiae* HDN21-0307; 5

Fig S3: HRESIMS spectrum of compound 1; 6

Fig S4: ^{1}H NMR spectrum (400 MHz, CD_3OD) of compound 1;...... 7

Fig S5: ^{13}C NMR spectrum (125 MHz, CD_3OD) of compound 1; 7

Fig S6: HSQC spectrum of compound 1; 8

Fig S7: HMBC spectrum of compound 1;...... 8

Fig S8: COSY spectrum of compound 1;...... 9

Fig S9: IR spectrum of compound 1;...... 9

Fig S10: HRESIMS spectrum of compound 2;...... 10

Fig S11: ^{1}H NMR spectrum (400 MHz, $CDCl_3$) of compound 2;...... 10

Fig S12: ^{13}C NMR spectrum (125 MHz, $CDCl_3$) of compound 2;...... 11

Fig S13: HSQC spectrum of compound 2; 11

Fig S14: HMBC spectrum of compound 2;...... 12

Fig S15: COSY spectrum of compound 2;...... 12

Fig S16: NOESY spectrum of compound 2; 13

Fig S17: HSQMBC spectrum of compound 2; 13

Fig S18: $^{3}J_{C\text{-}H}$ values obtained from the HSQMBC spectrum (500 MHz, $CDCl_3$) of 2. 14

Fig S19: IR spectrum of compound 2;...... 14

Fig S20: HRESIMS spectrum of compound 3;...... 15

Fig S21: ^{1}H NMR spectrum (500 MHz, Acetone-d_6) of compound 3;...... 15

Fig S22: ^{13}C NMR spectrum (125 MHz, Acetone-d_6) of compound 3;...... 16

Fig S23: HSQC spectrum of compound 3; 16

Fig S24: HMBC spectrum of compound 3;...... 17

Fig S25: COSY spectrum of compound 3;...... 17

Fig S26: NOESY spectrum of compound 3; 18

Fig S27: IR spectrum of compound 3;...... 18

Fig S28: HRESIMS spectrum of compound 4;...... 19

Fig S29: ^{1}H NMR spectrum (500 MHz, DMSO-d_6) of compound 4; 19

Fig S30: ^{13}C NMR spectrum (125 MHz, DMSO-d_6) of compound 4; **20**

Fig S31: HSQC spectrum of compound 4; **20**

Fig S32: HMBC spectrum of compound 4; **21**

Fig S33: COSY spectrum of compound 4; **21**

Fig S34: NOESY spectrum of compound 4; **22**

Fig S35: IR spectrum of compound 4; **22**

Fig S36: HRESIMS spectrum of compound 5; **23**

Fig S37: ^{1}H NMR spectrum (400 MHz, $CDCl_3$) of compound 5; **23**

Fig S38: ^{13}C NMR spectrum (100 MHz, $CDCl_3$) of compound 5; **24**

Fig S39: HSQC spectrum of compound 5; **24**

Fig S40: HMBC spectrum of compound 5; **25**

Fig S41: COSY spectrum of compound 5; **25**

Fig S42: NOESY spectrum of compound 5; **26**

Fig S43: HSQMBC spectrum of compound 5; **26**

Fig S44: $^{3}J_{C-H}$ values obtained from the HSQMBC spectrum (400 MHz, $CDCl_3$) of 5. .... **27**

Fig S45: IR spectrum of compound 5; **27**

Fig S46: HRESIMS spectrum of compound 6; **28**

Fig S47: ^{1}H NMR spectrum (100 MHz, CD_3OD) of compound 6; **28**

Fig S48: ^{13}C NMR spectrum (100 MHz, CD_3OD) of compound 6; **29**

Fig S49: HSQC spectrum of compound 6; **29**

Fig S50: HMBC spectrum of compound 6; **30**

Fig S51: COSY spectrum of compound 6; **30**

Fig S52: NOESY spectrum of compound 6; **31**

Fig S53: IR spectrum of compound 6; **31**

CD Calculation **31**

Fig S54: ECD: B3LYP/6-31G(d) optimized lowest energy conformers for 3; **32**

NMR Calculation **32**

Fig S55: B3LYP/6-31G(d) optimized lowest energy conformers for 3; **33**

Fig S56: NMR calculations with DP4+ probability analysis for compounds 3. .... **33**

Table S2. DP4+ evaluation of theoretical and experimental one of 3. .... **33**

Fig S57: B3LYP/6-31G(d) optimized lowest energy conformers for 4; **35**

Fig S58: NMR calculations with DP4+ probability analysis for compounds 4. .... **35**

Table S3. DP4+ evaluation of theoretical and experimental one of 4. .... **35**

References.. **37**

Fig S1: OSMAC cultivation strategy for *T. amestolkiae* HDN21-0307;

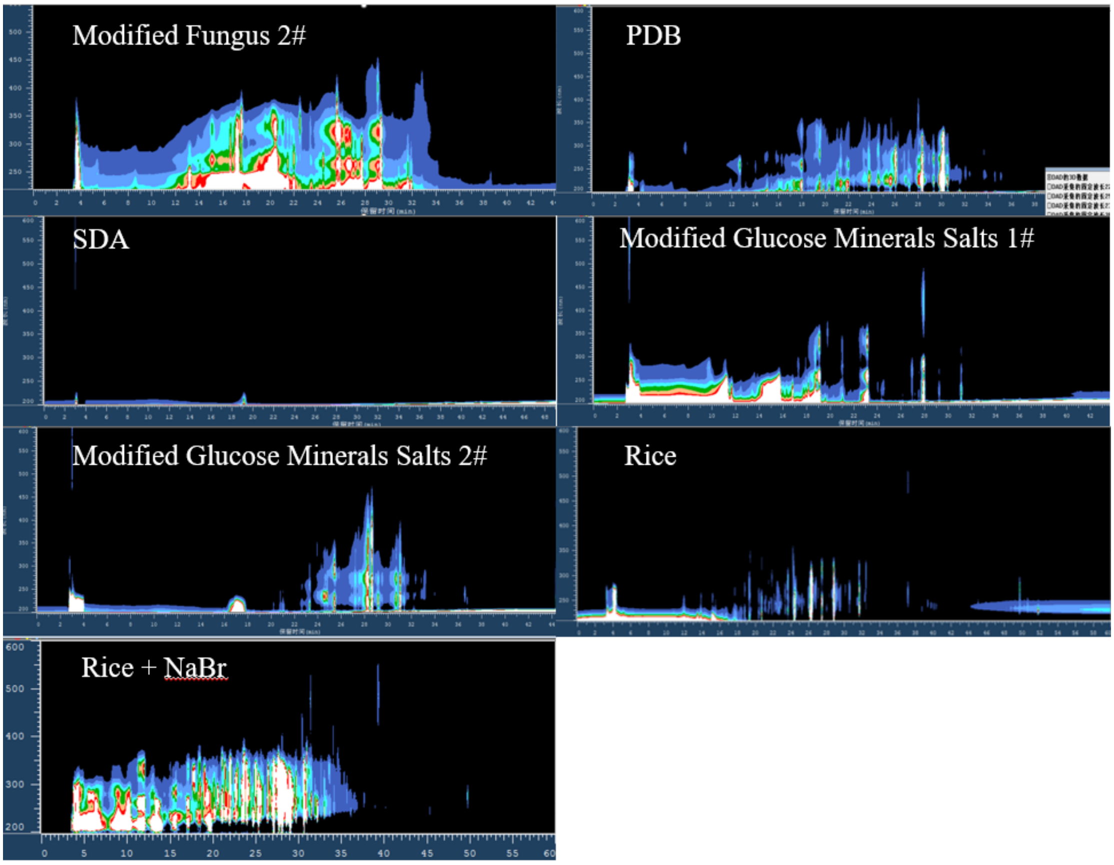

Table S1: OSMAC consists of media for *T. amestolkiae* HDN21-0307;

Name	Media composition
N1	glucose 4%, peptone 1%, monosodium glutamate 1%, cysteine 0.3%, KH_2PO4 0.05%, MgSO4·7H2O 0.03%, corn steep liquor 0.1% and yeast extract 0.3%, seawater
N2	glucose 20 g/L, potato extract 200 g/L, seawater
N3	peptone 1%, NaCl 0.5%, glucose 4%, seawater
N4	(N-Acetyl-D-glucosamine 3.2%, $NaNO_3$ 1%, K_2HPO_4 0.006%, $MgSO_4{\cdot}7H_2O$ 1%, $MnSO_4$ 0.006%, $Fe_2(SO_4)_3$ 0.006%, $CaCl_2$ 0.006%, seawater
N5	glucose 3.2%, $NaNO_3$ 1%, K_2HPO_4 0.006%, $MgSO_4{\cdot}7H_2O$ 1%, $MnSO_4$ 0.006%, $Fe_2(SO_4)_3$ 0.006%, $CaCl_2$ 0.006%, seawater
N6	rice 80 g, freshwater 120 ml
N7	NaBr 1 g, rice 80 g, freshwater 120 ml

Fig S2: The 16S rRNA sequences data of *T. amestolkiae* HDN21-0307;

CCCTTGTCTCTATACACCTGTTGCTTTGGCGGGCCCACCGGGGCCACCTGGTC

GCCGGGGGACATCTGTCCCCGGGCCCGCGCCCGCCGAAGCGCTCTGTGAACC

CTGATGAAGATGGGCTGTCTGAGTACTATGAAAATTGTCAAAACTTTCAACAA

TGGATCTCTTGGTTCCGGCATCGATGAAGAACGCAGCGAAATGCGATAAGTA

ATGTGAATTGCAGAATTCCGTGAATCATCGAATCTTTGAACGCACATTGCGCC

CCCTGGCATTCCGGGGGGCATGCCTGTCCGAGCGTCATTTCTGCCCTCAAGCA

CGGCTTGTGTGTTGGGTGCGGTCCCCCCGGGGACCTGCCCGAAAGGCAGCGG

CGACGTCCGTCTGGTCCTCGAGCGTATGGGGCTTTGTCACTCGCTCGGGAAGG

ACTGGCGGGGGTTGGTCACCACCAAAATTTTACCACGGTTGACCTCGGATCA

GGTAGGAGTTACCCGCTGAACTTAAGCATATCAATAA

Fig S3: HRESIMS spectrum of compound 1;

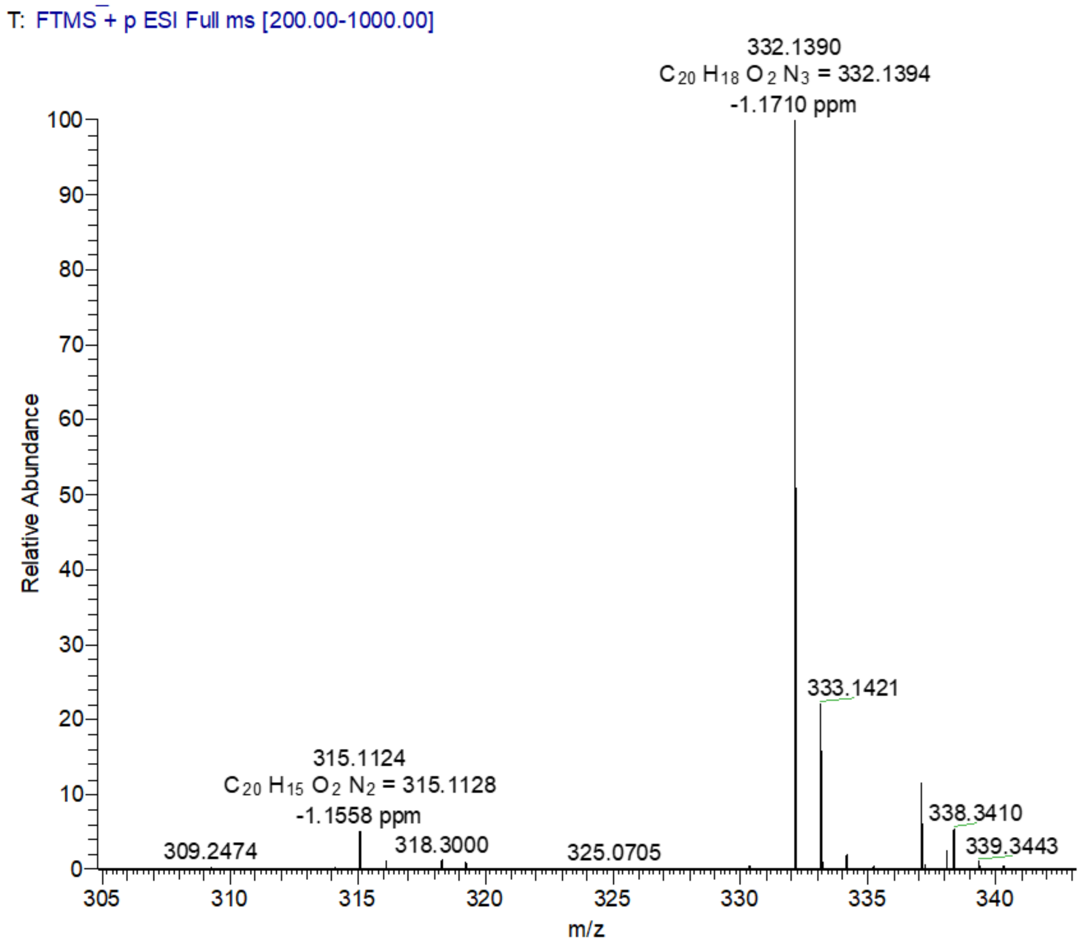

Fig S4: ^{1}H NMR spectrum (400 MHz, CD_3OD) of compound 1;

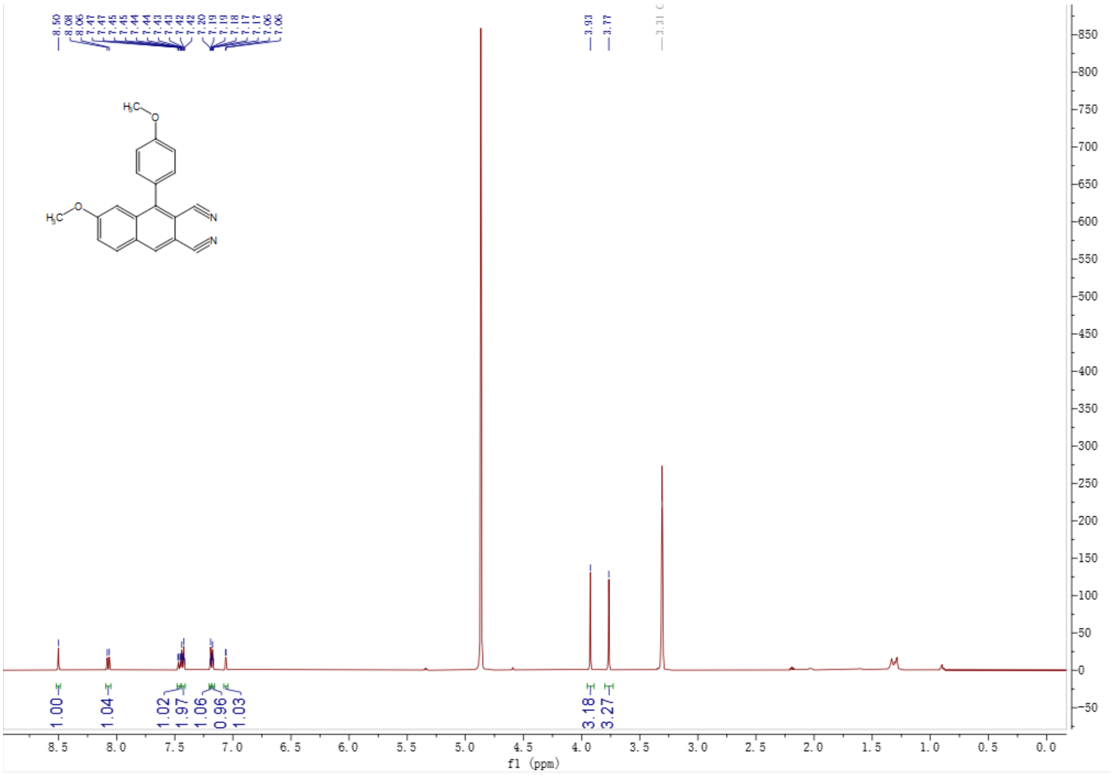

Fig S5: ^{13}C NMR spectrum (125 MHz, CD_3OD) of compound 1;

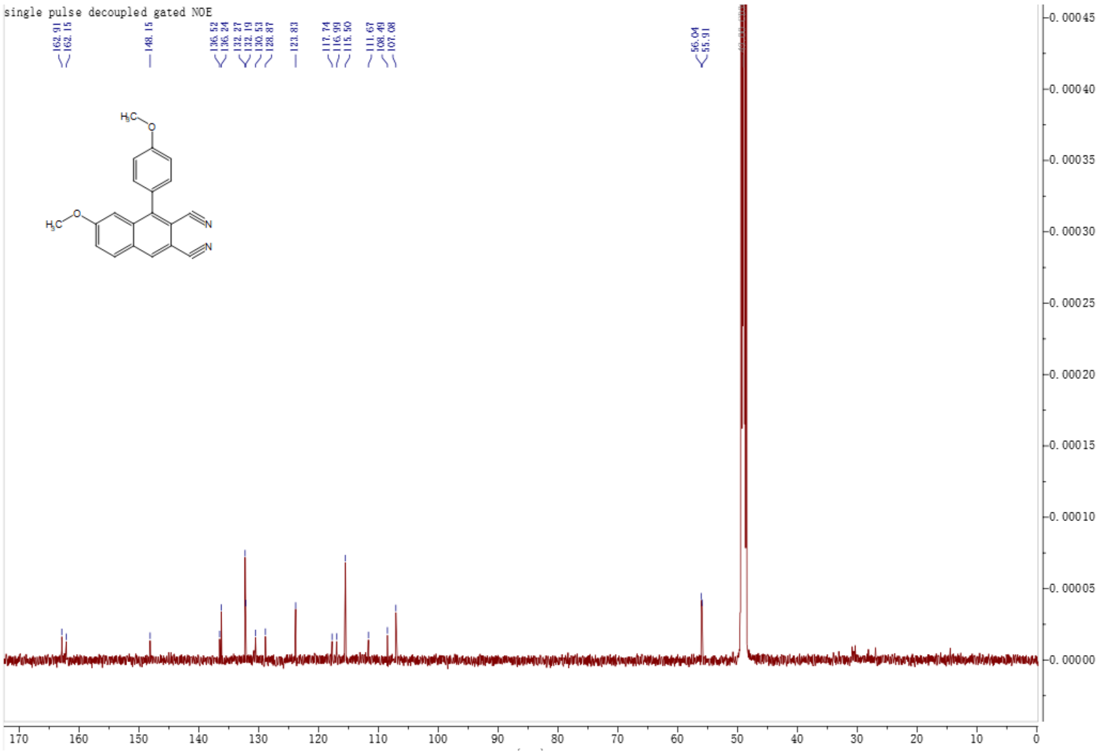

Fig S6: HSQC spectrum of compound 1;

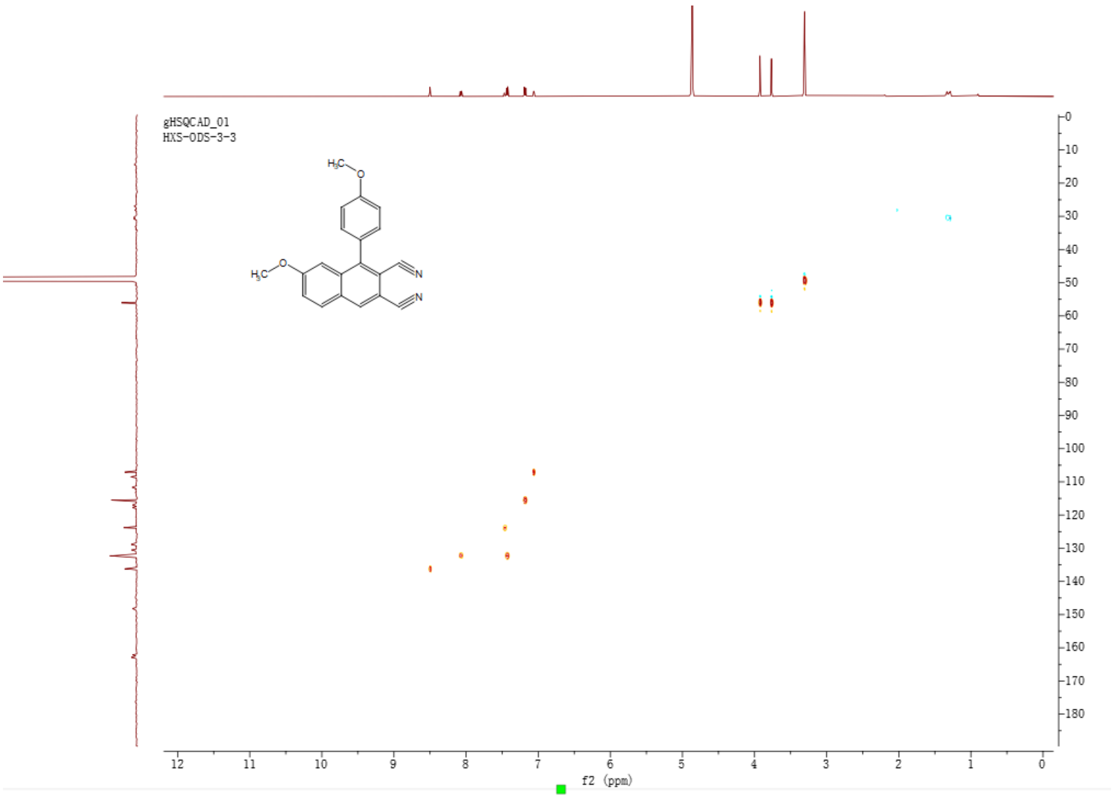

Fig S7: HMBC spectrum of compound 1;

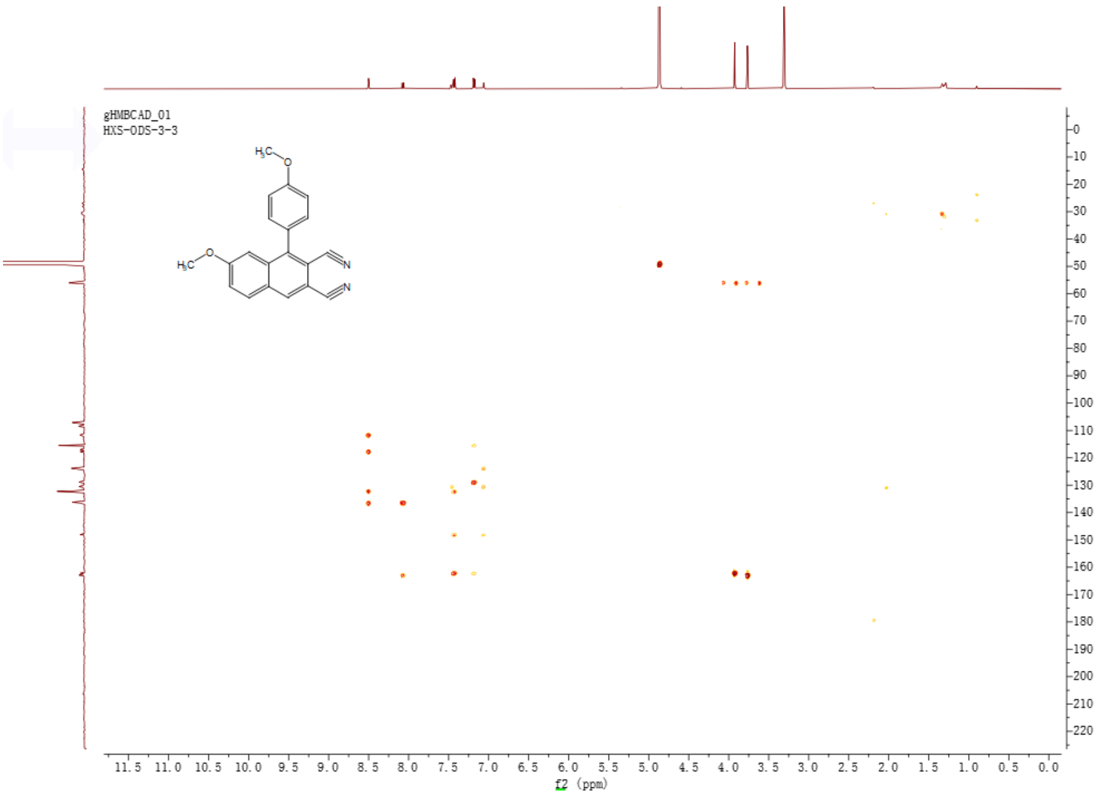

Fig S8: COSY spectrum of compound 1;

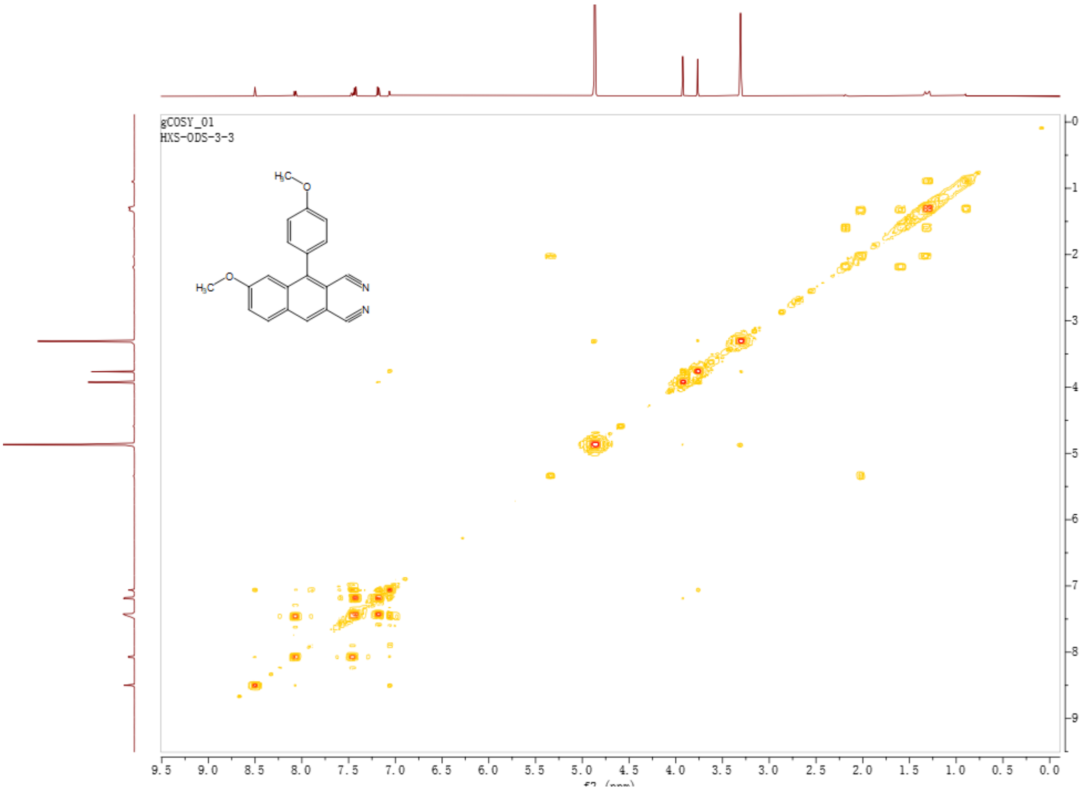

Fig S9: IR spectrum of compound 1;

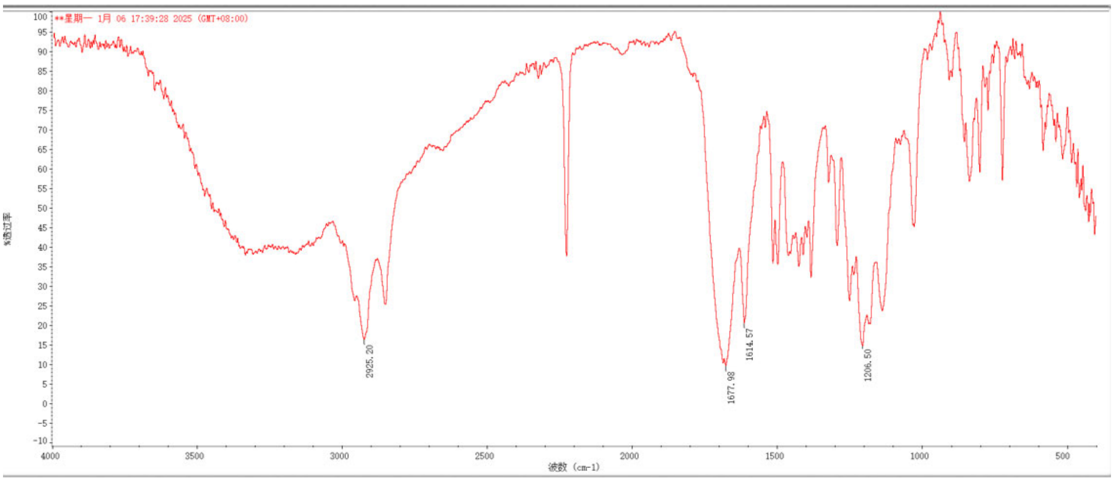

Fig S10: HRESIMS spectrum of compound 2;

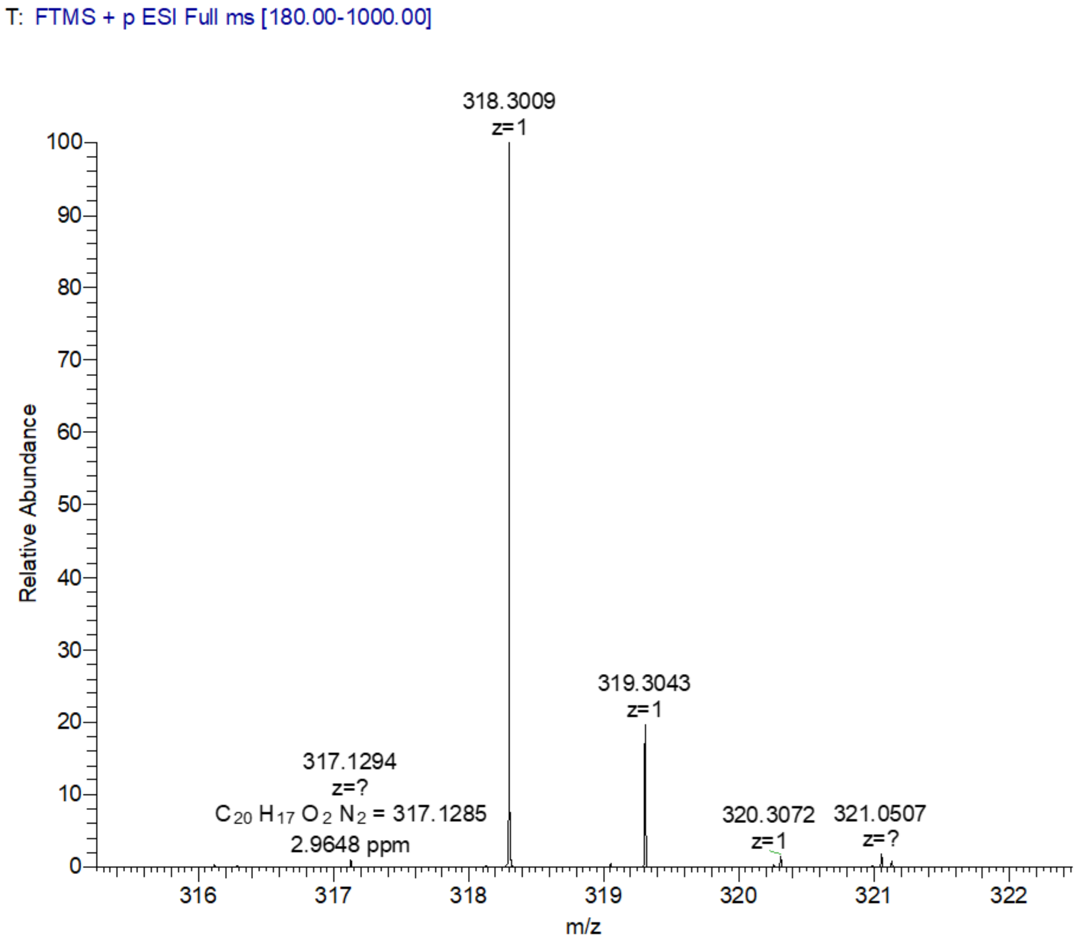

Fig S11: ^{1}H NMR spectrum (400 MHz, CDCl₃) of compound 2;

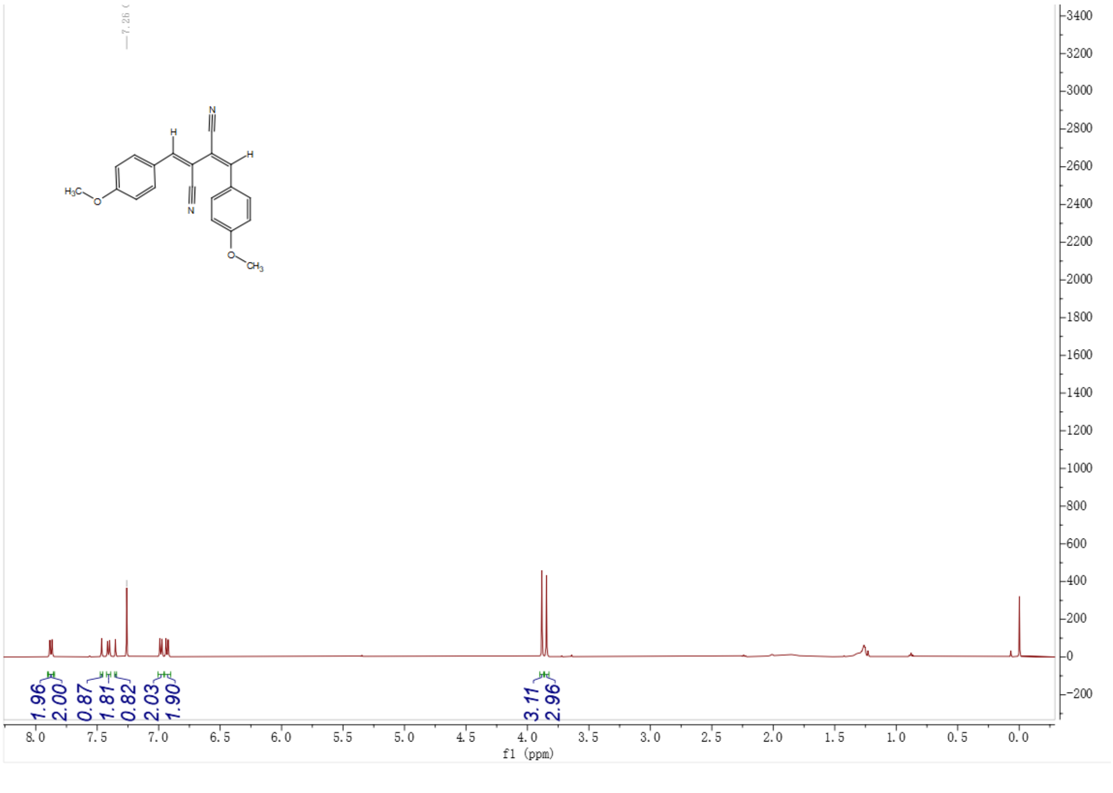

Fig S12: ^{13}C NMR spectrum (125 MHz, $CDCl_3$) of compound 2;

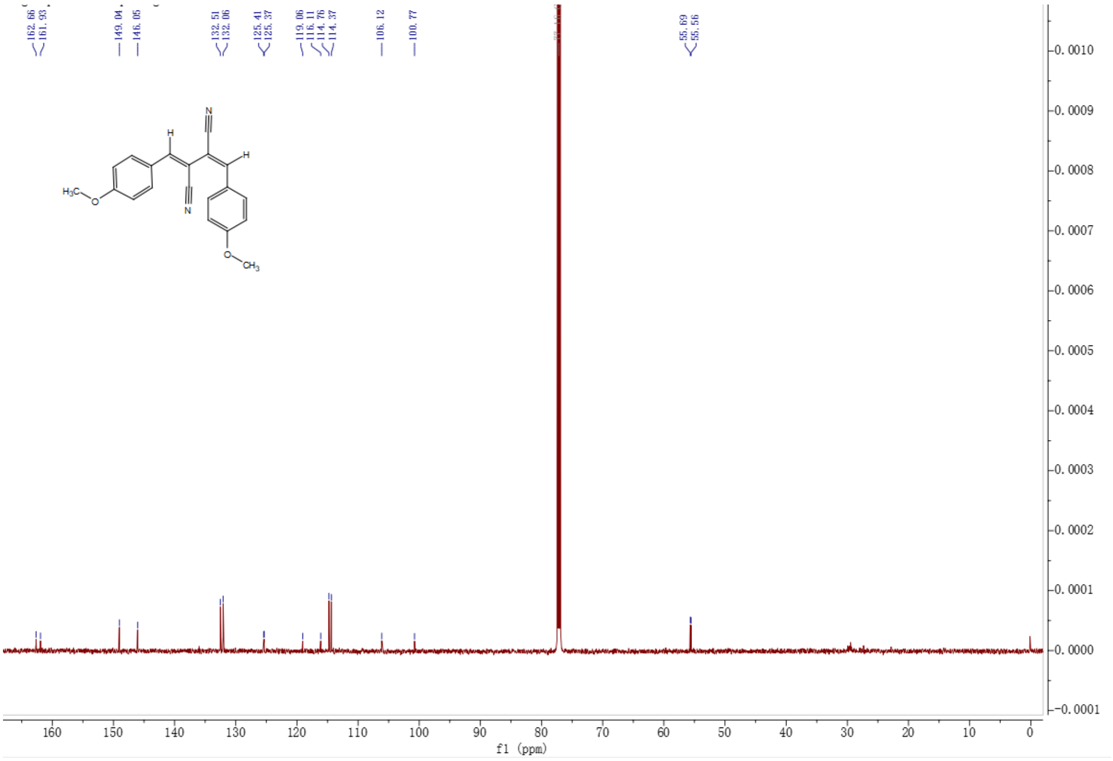

Fig S13: HSQC spectrum of compound 2;

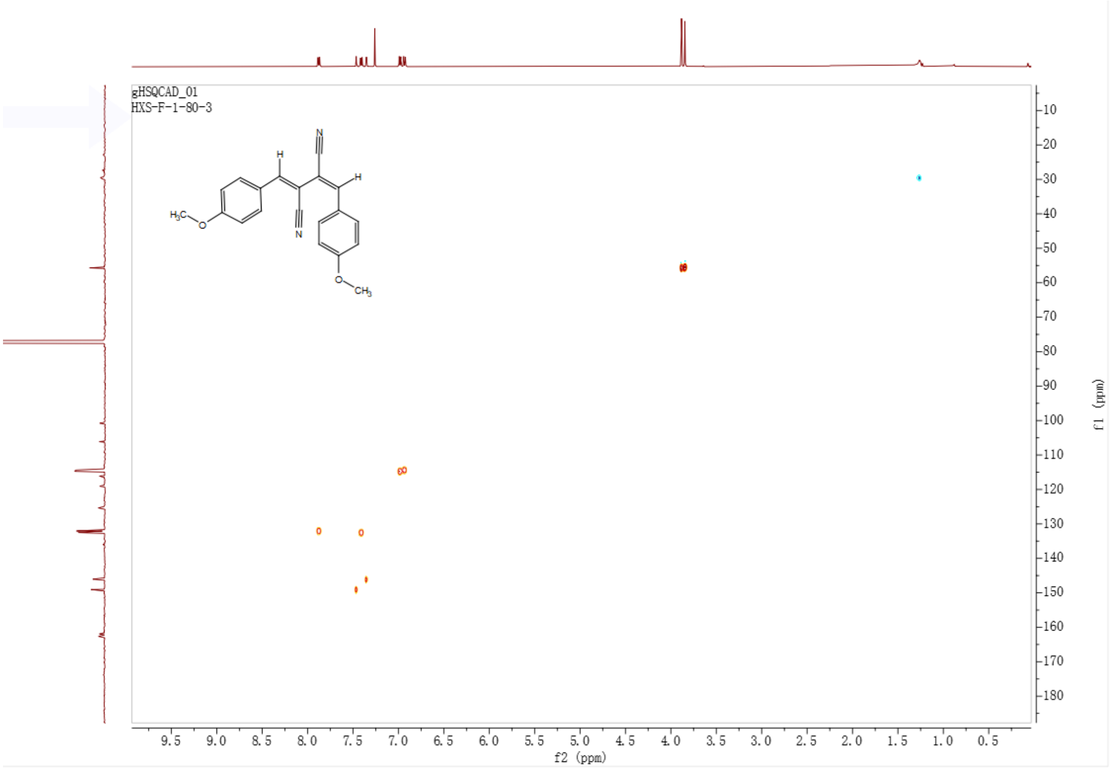

Fig S14: HMBC spectrum of compound 2;

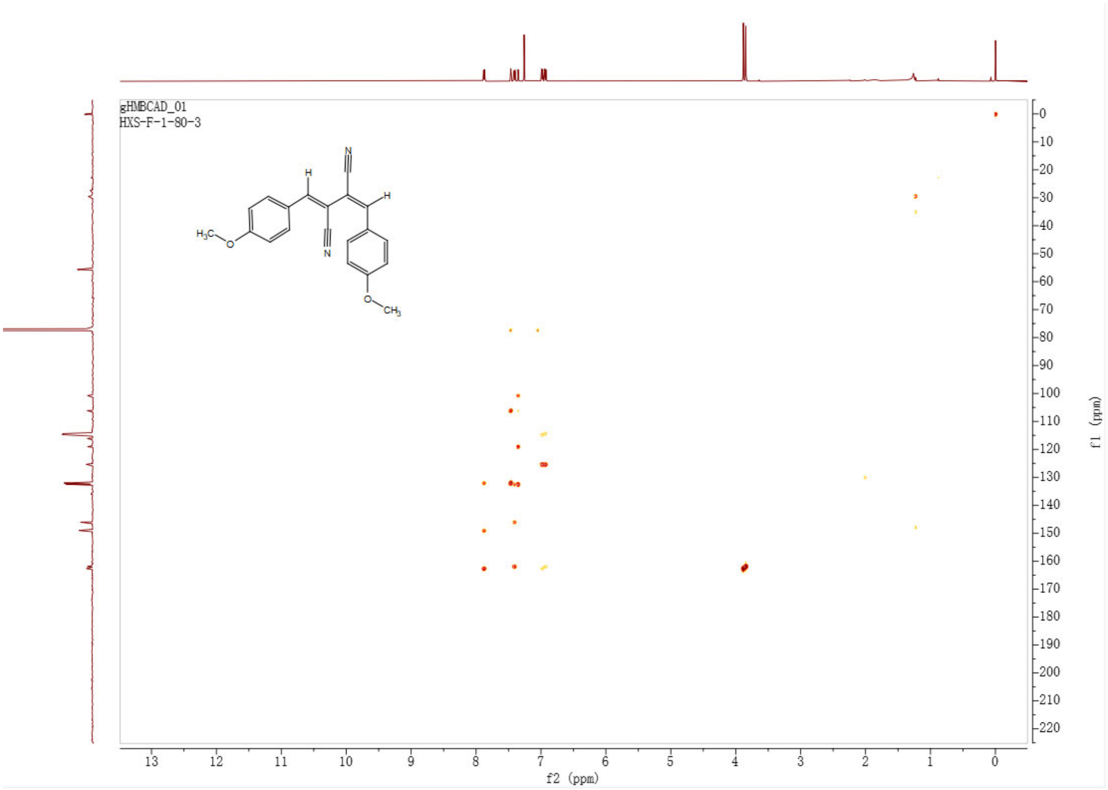

Fig S15: COSY spectrum of compound 2;

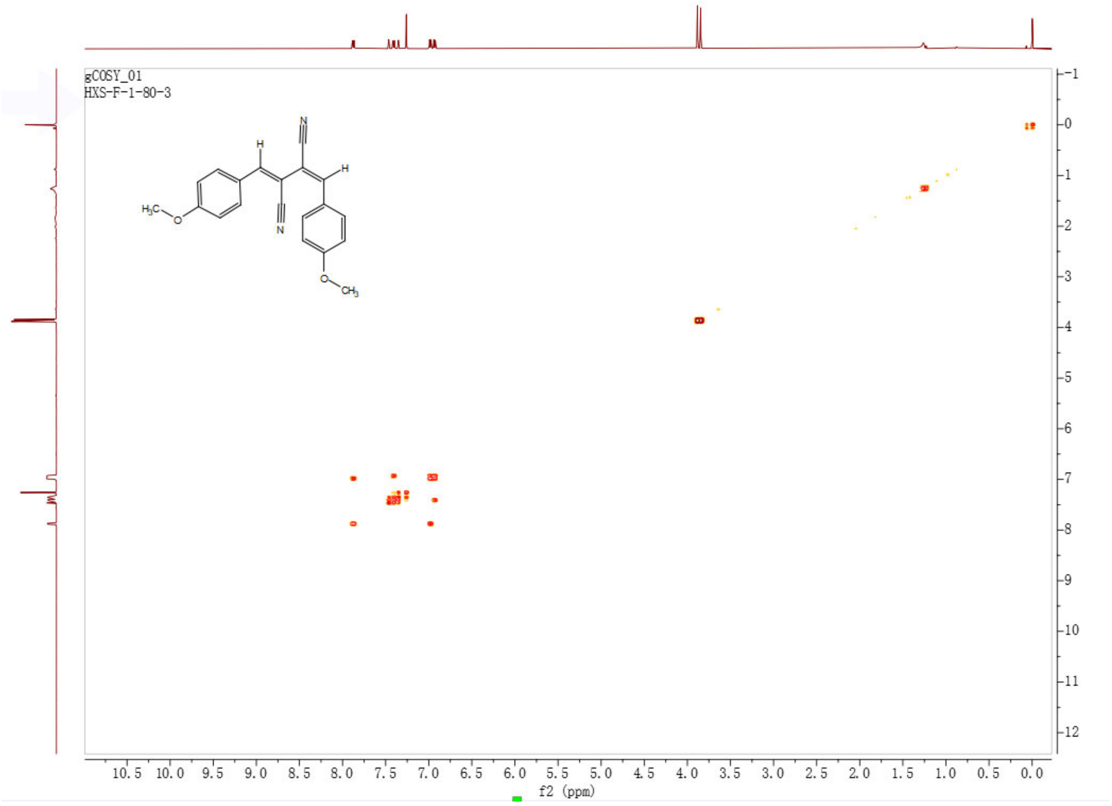

Fig S16: NOESY spectrum of compound 2;

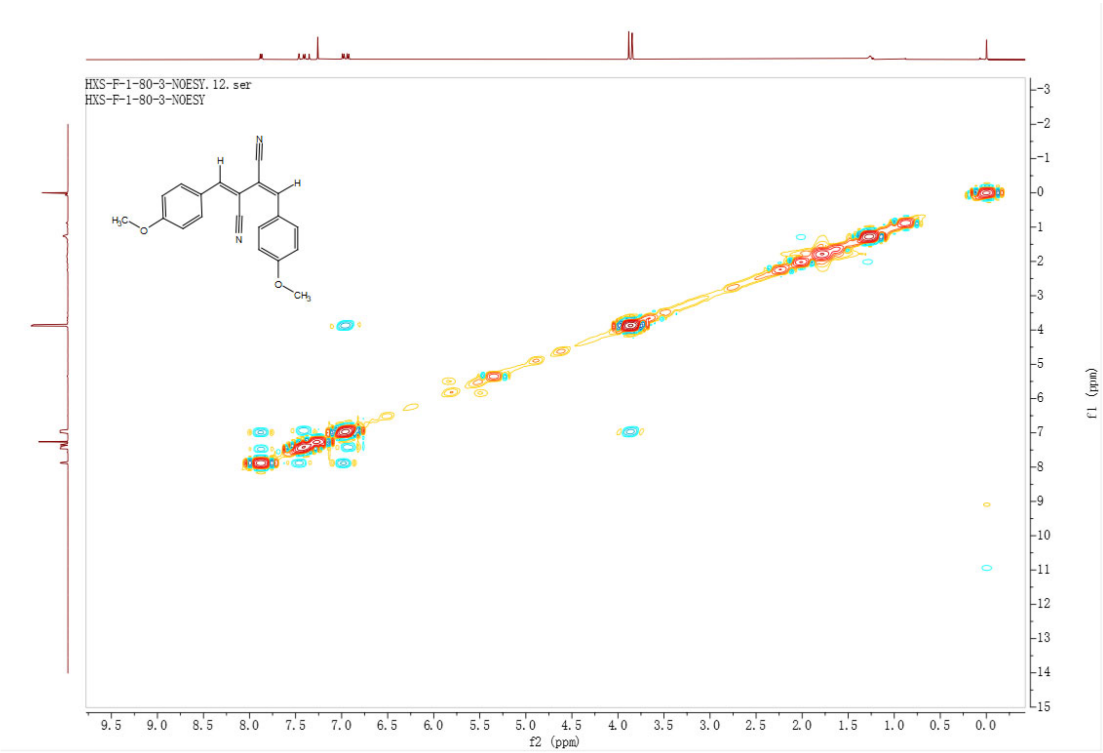

Fig S17: HSQMBC spectrum of compound 2;

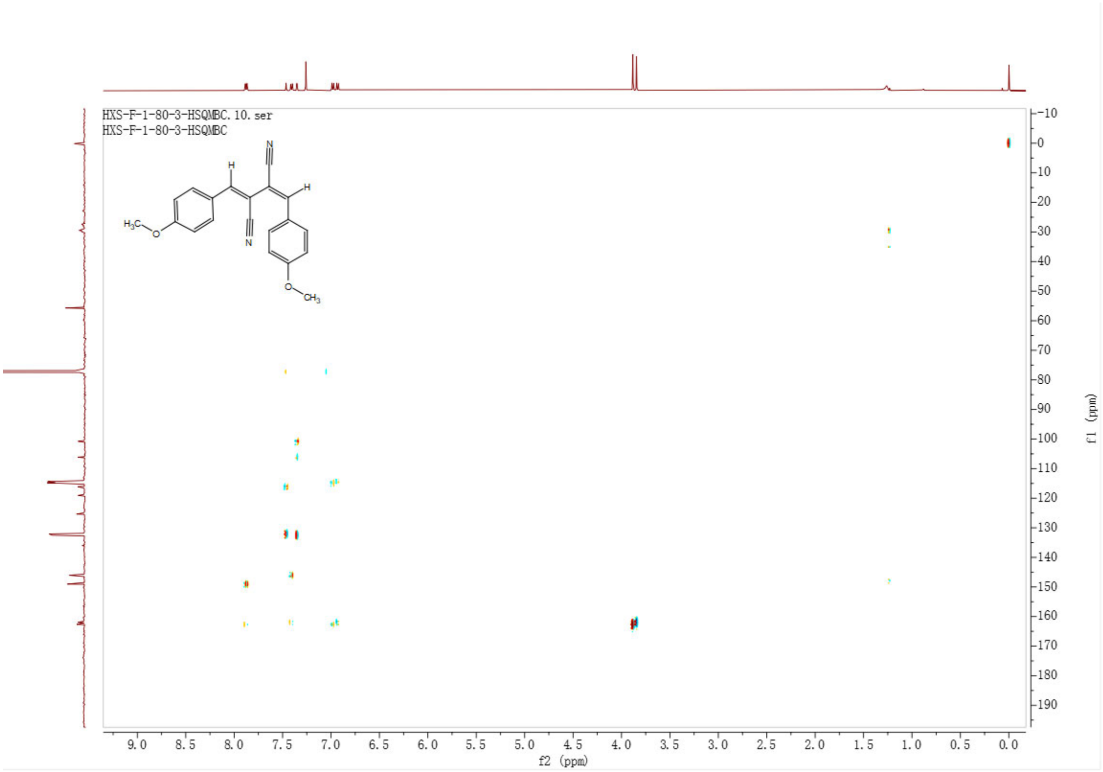

Fig S18: $^{3}J_{C\text{-}H}$ values obtained from the HSQMBC spectrum (500 MHz, $CDCl_3$) of 2.

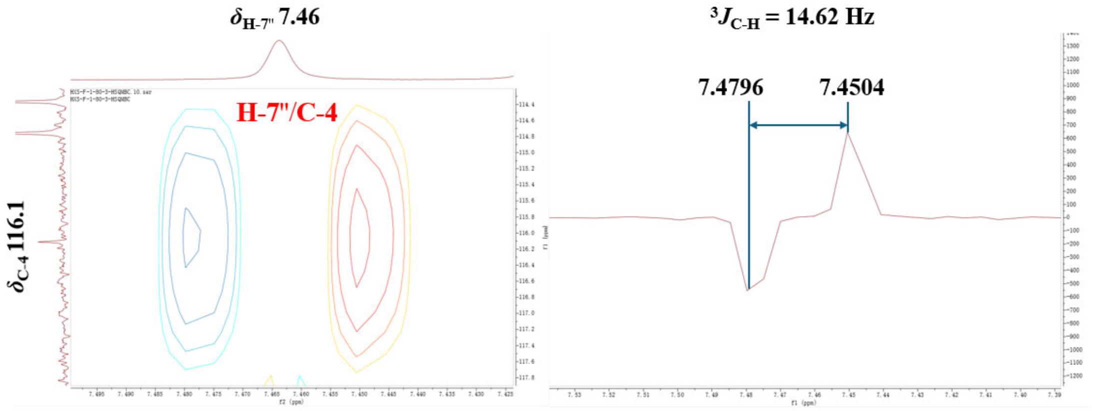

determined in CD_3OD

Fig S19: IR spectrum of compound 2;

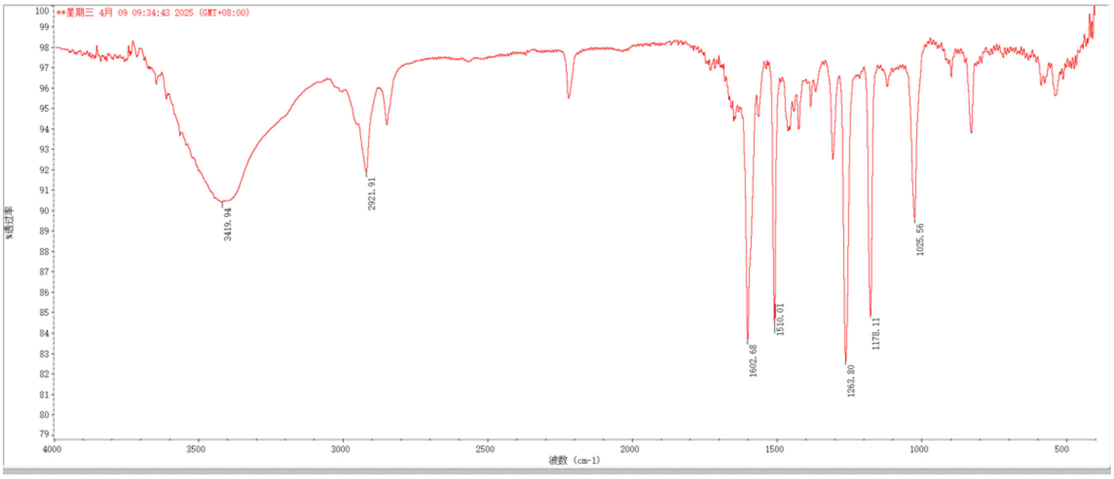

Fig S20: HRESIMS spectrum of compound 3;

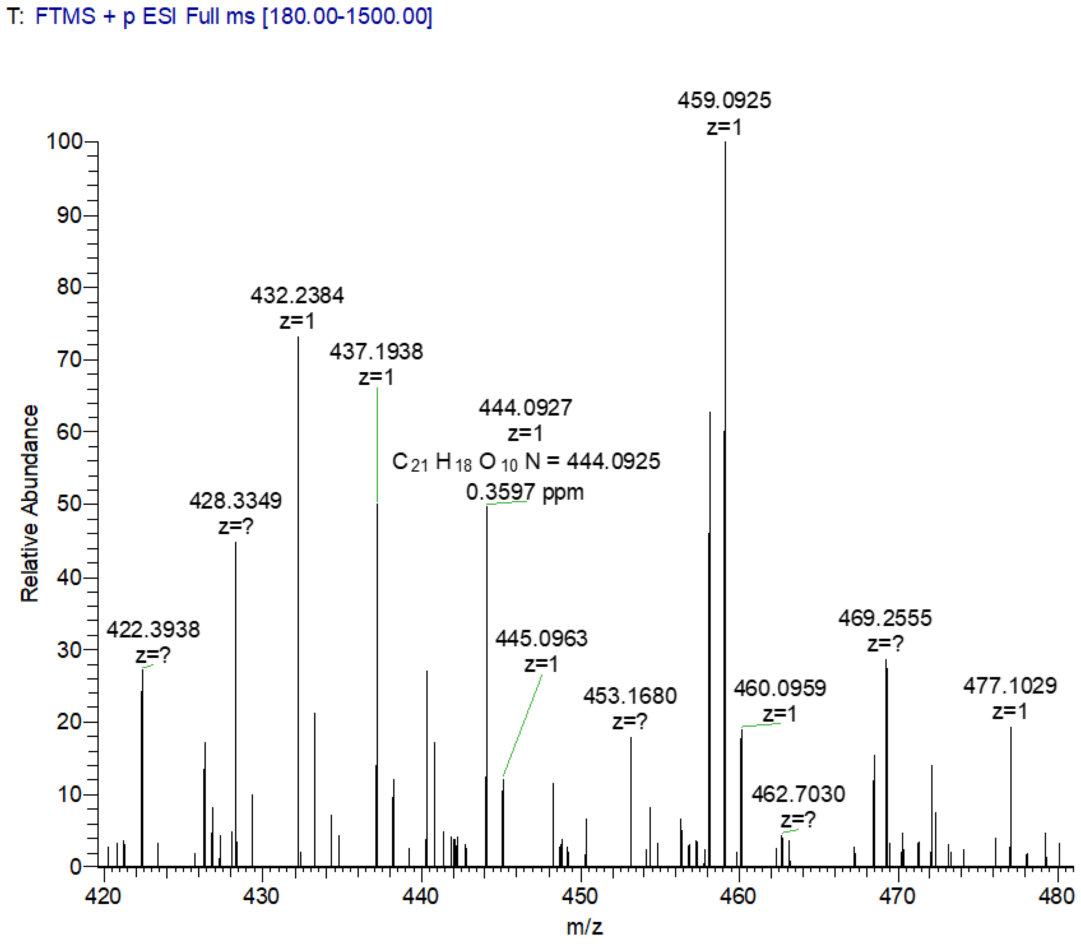

Fig S21: ^{1}H NMR spectrum (500 MHz, Acetone-d_6) of compound 3;

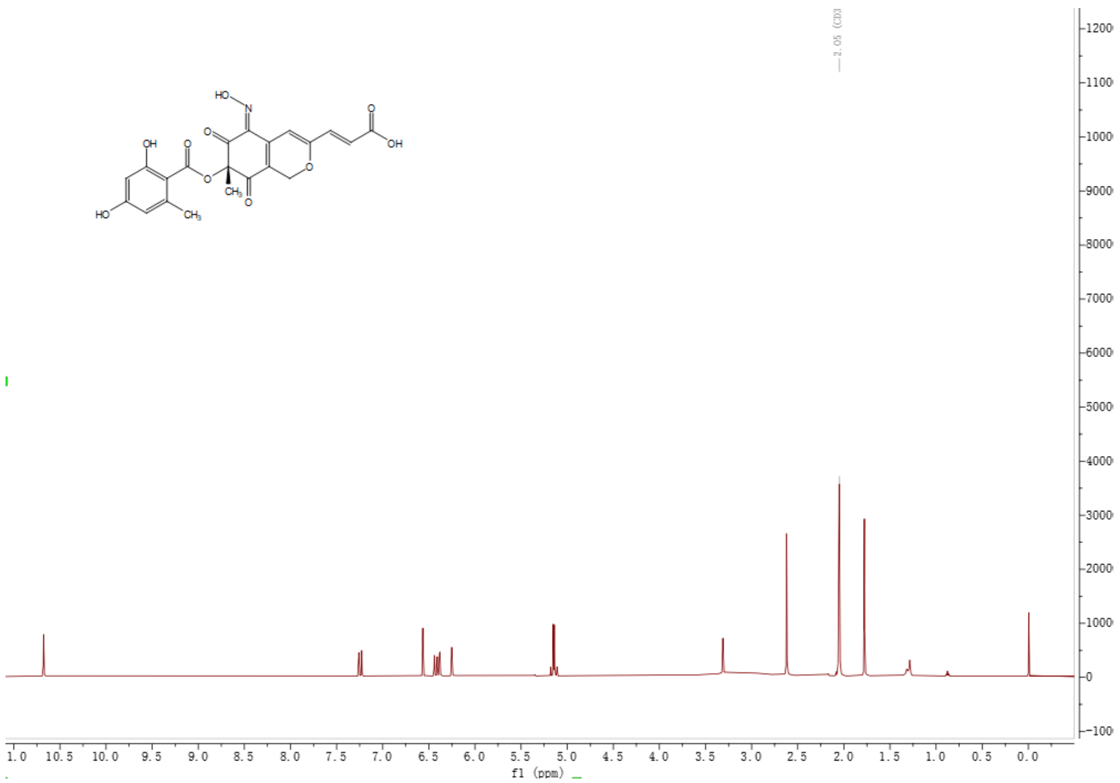

Fig S22: ^{13}C NMR spectrum (125 MHz, Acetone-d_6) of compound 3;

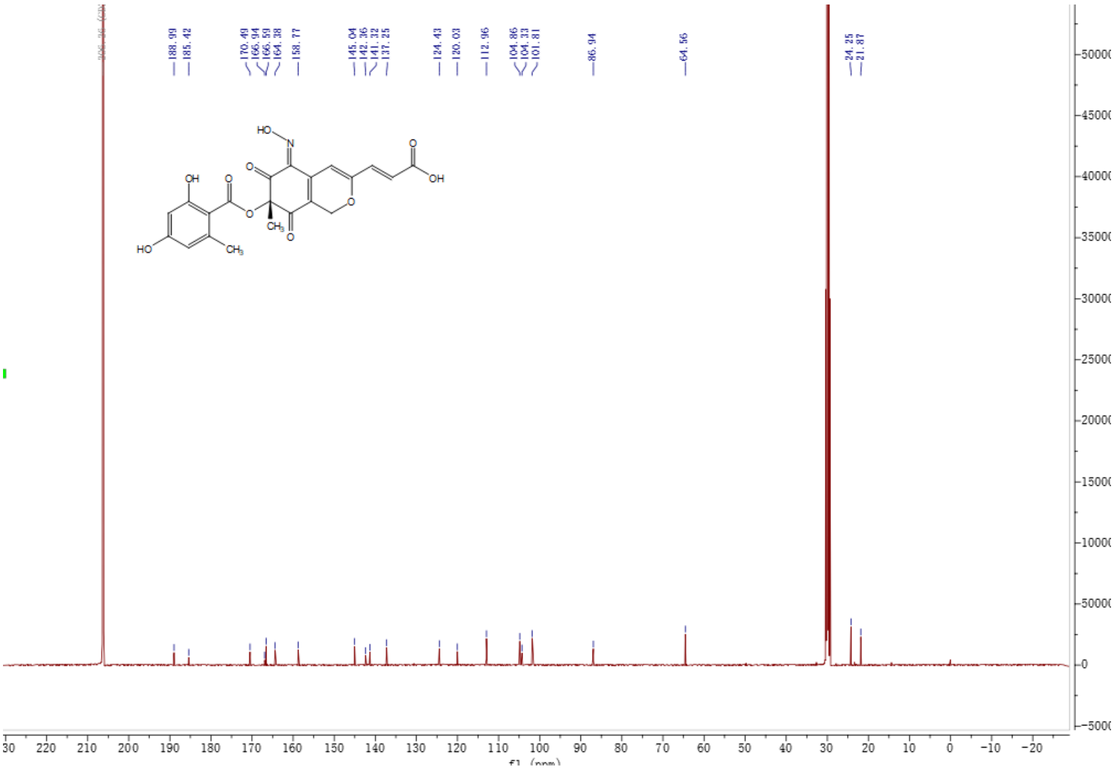

Fig S23: HSQC spectrum of compound 3;

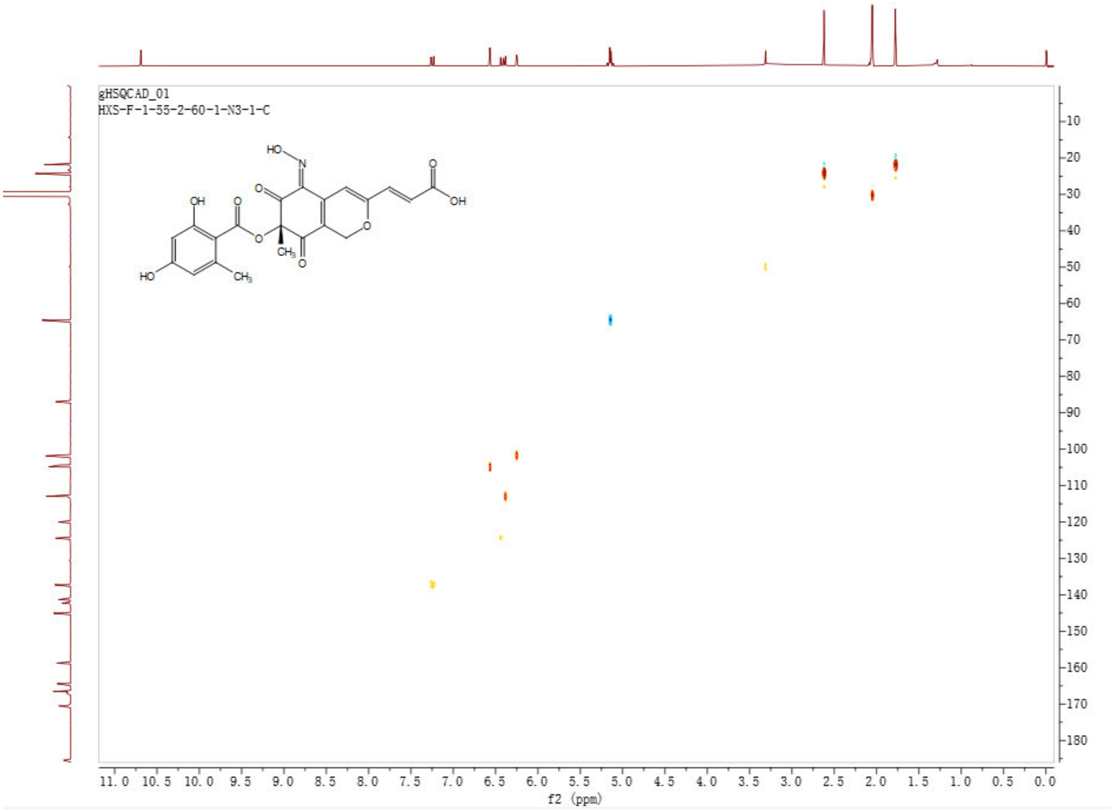

Fig S24: HMBC spectrum of compound 3;

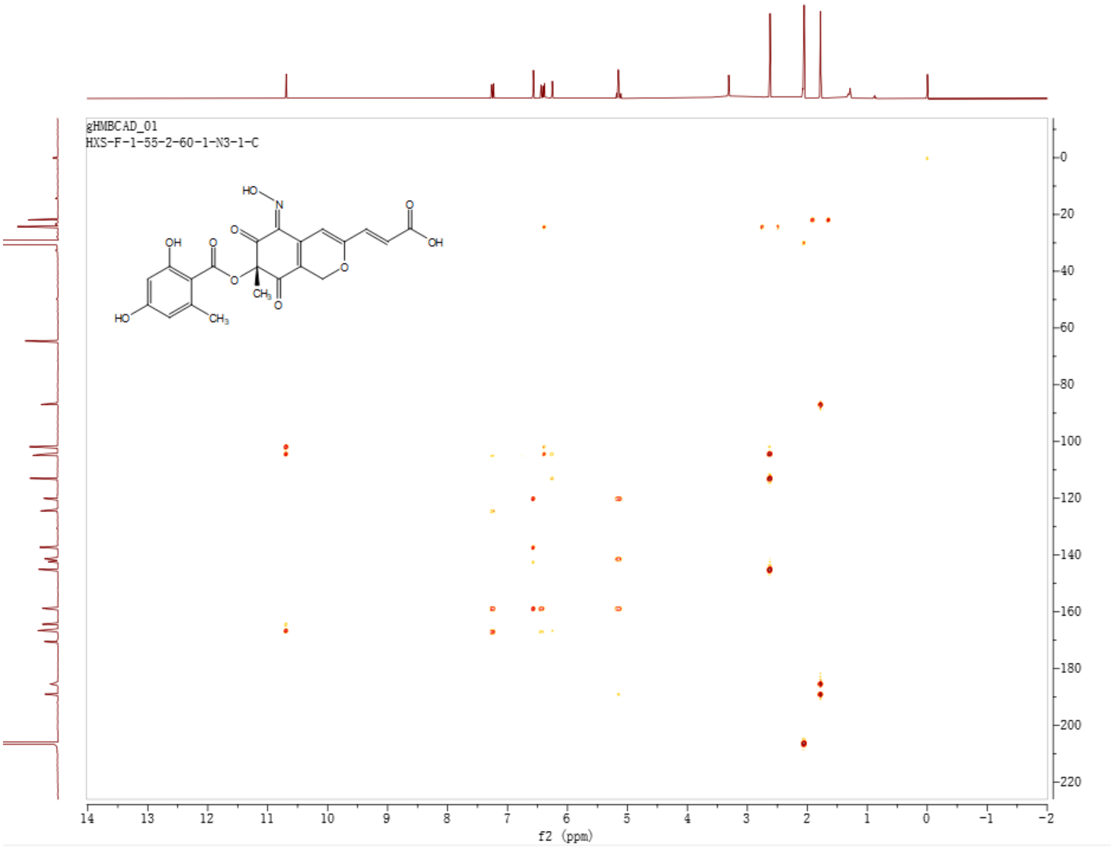

Fig S25: COSY spectrum of compound 3;

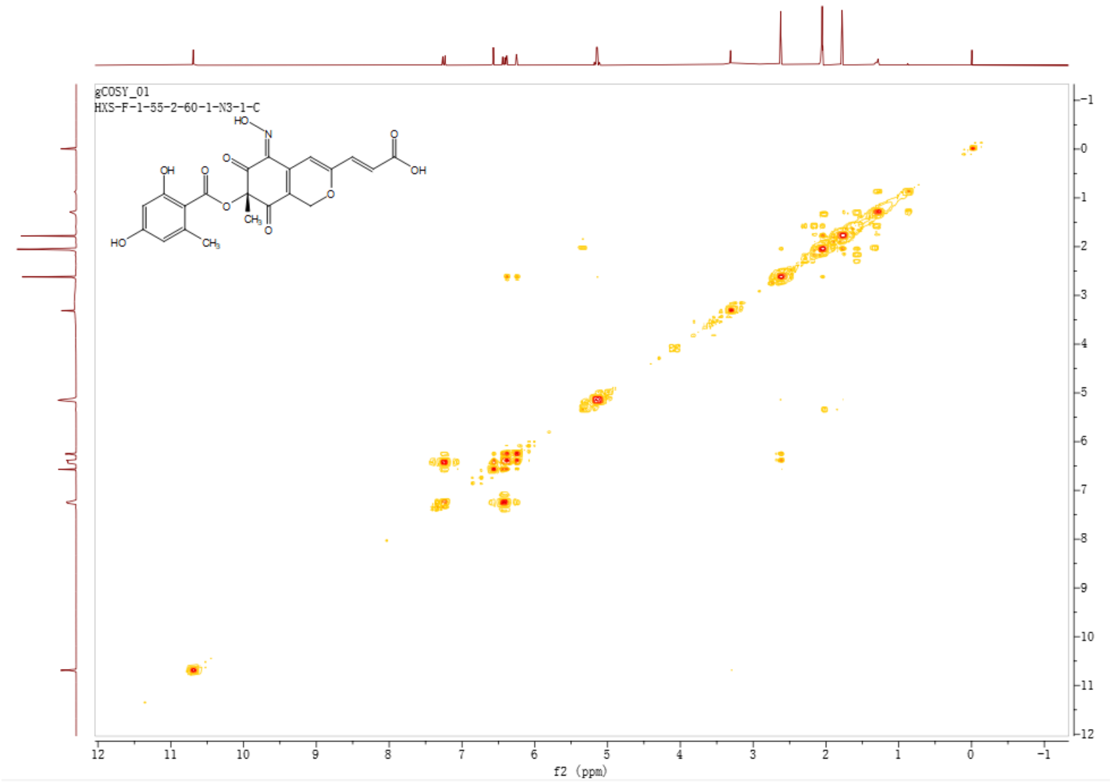

Fig S26: NOESY spectrum of compound 3;

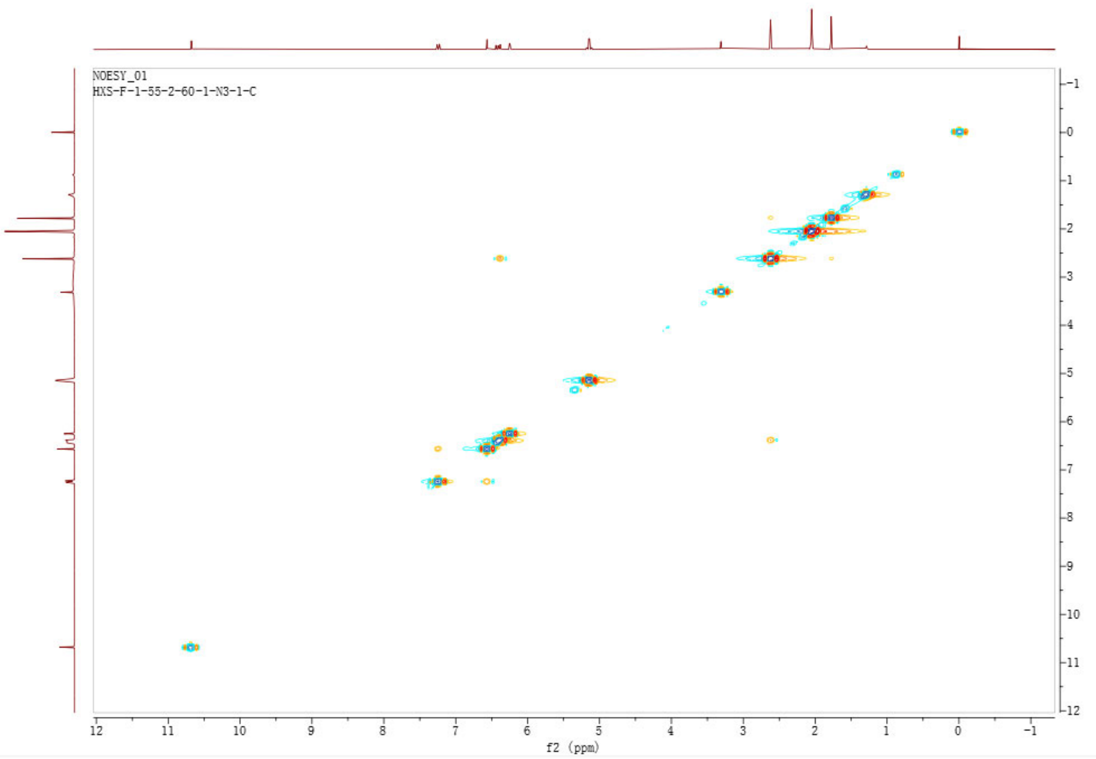

Fig S27: IR spectrum of compound 3;

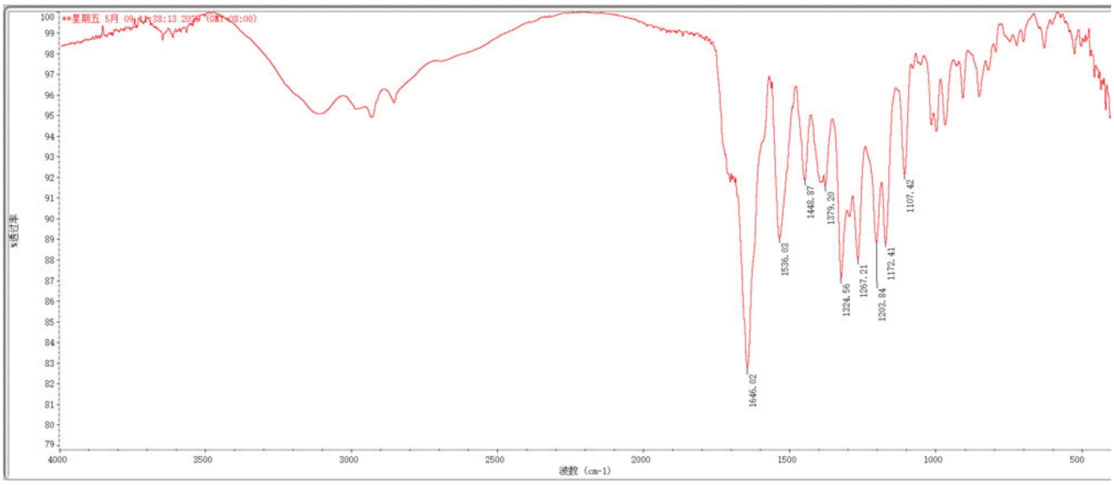

Fig S28: HRESIMS spectrum of compound 4;

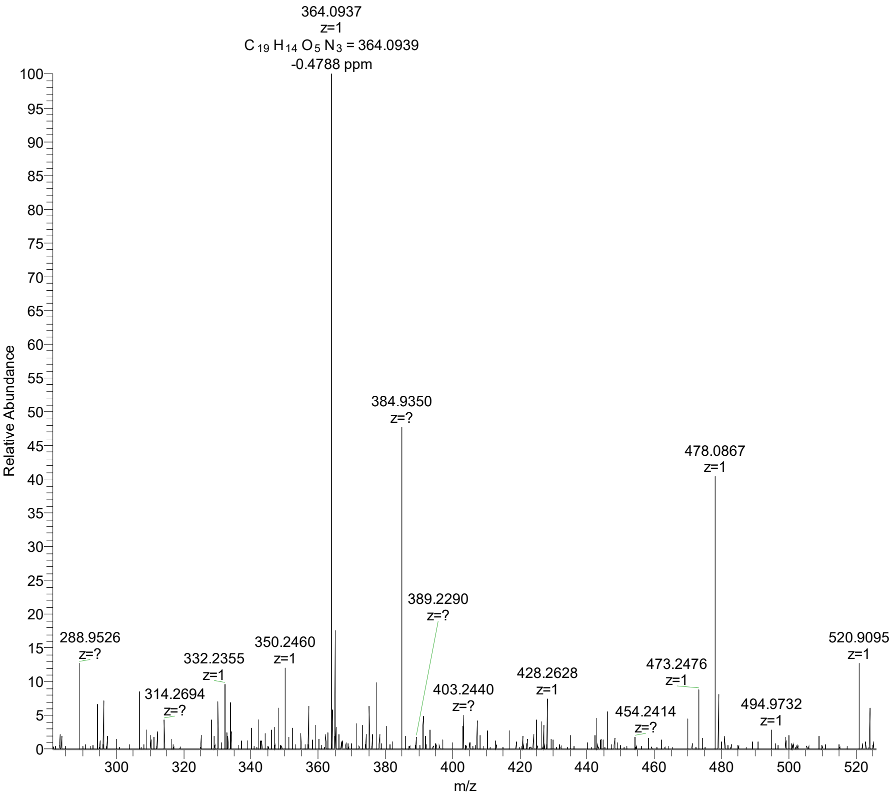

Fig S29: ^{1}H NMR spectrum (500 MHz, DMSO-*d*$_6$) of compound 4;

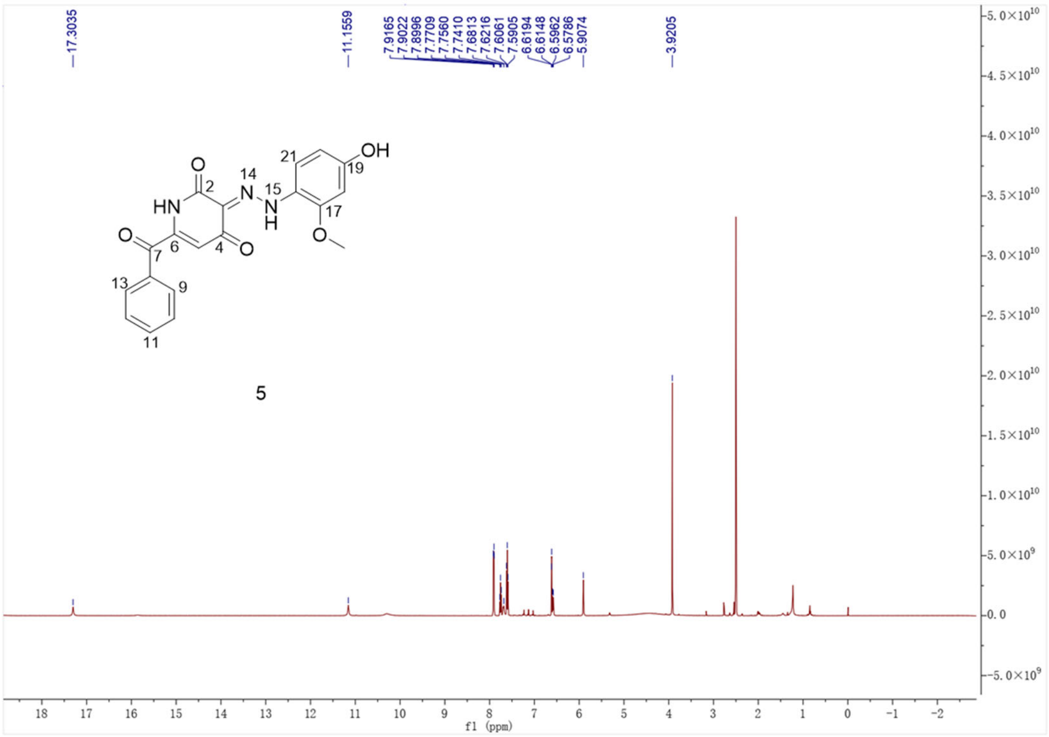

Fig S30: ^{13}C NMR spectrum (125 MHz, DMSO-d_6) of compound 4;

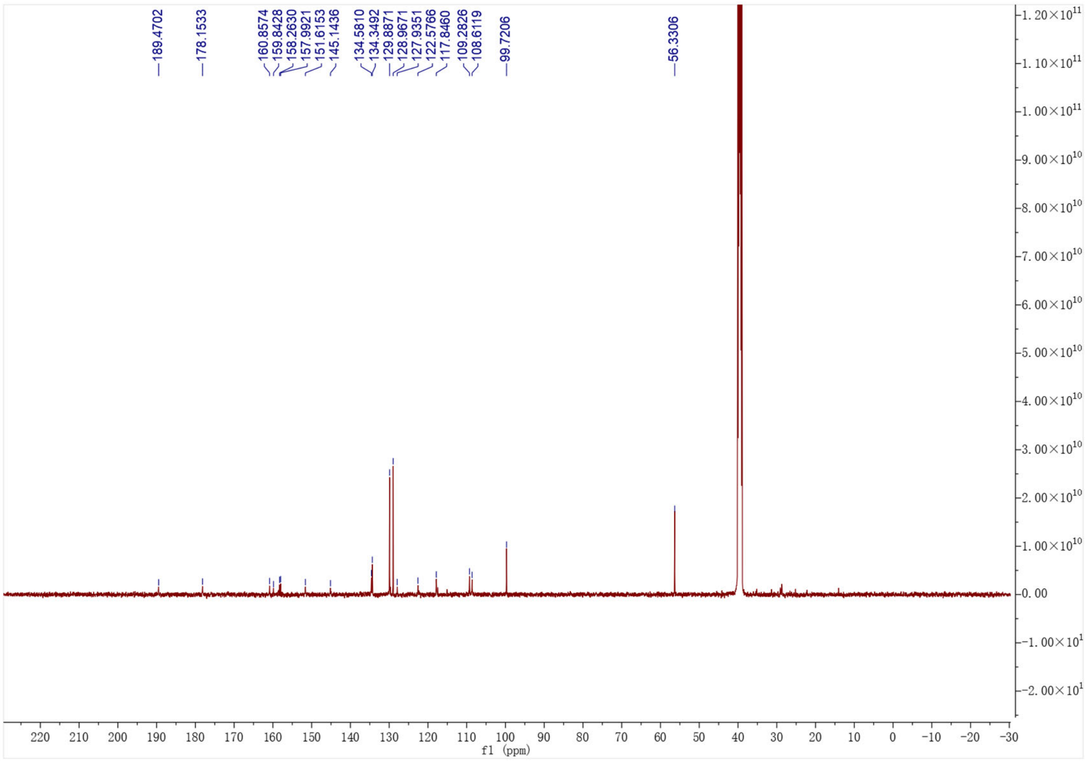

Fig S31: HSQC spectrum of compound 4;

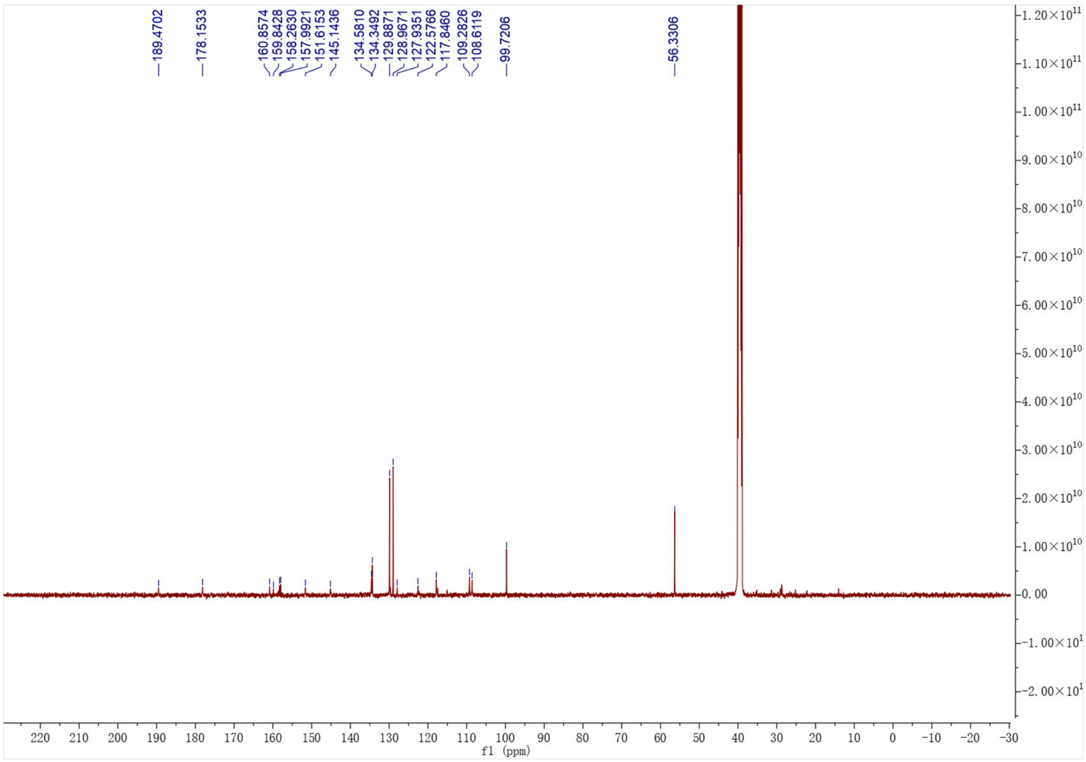

Fig S32: HMBC spectrum of compound 4;

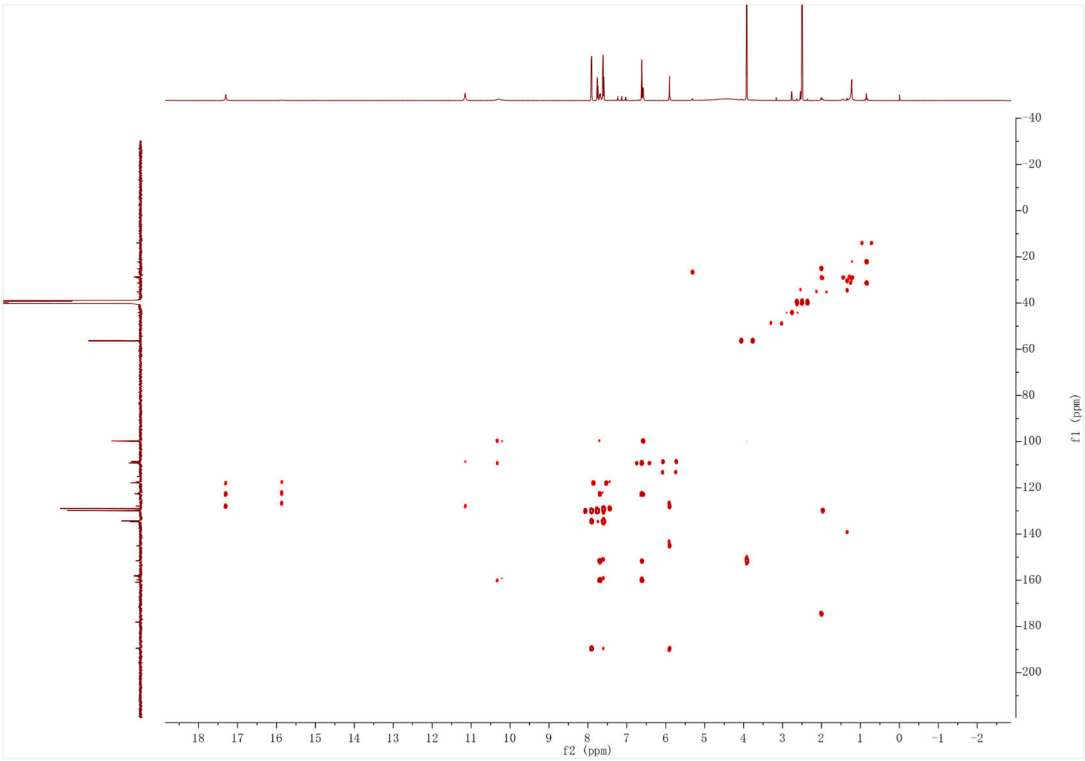

Fig S33: COSY spectrum of compound 4;

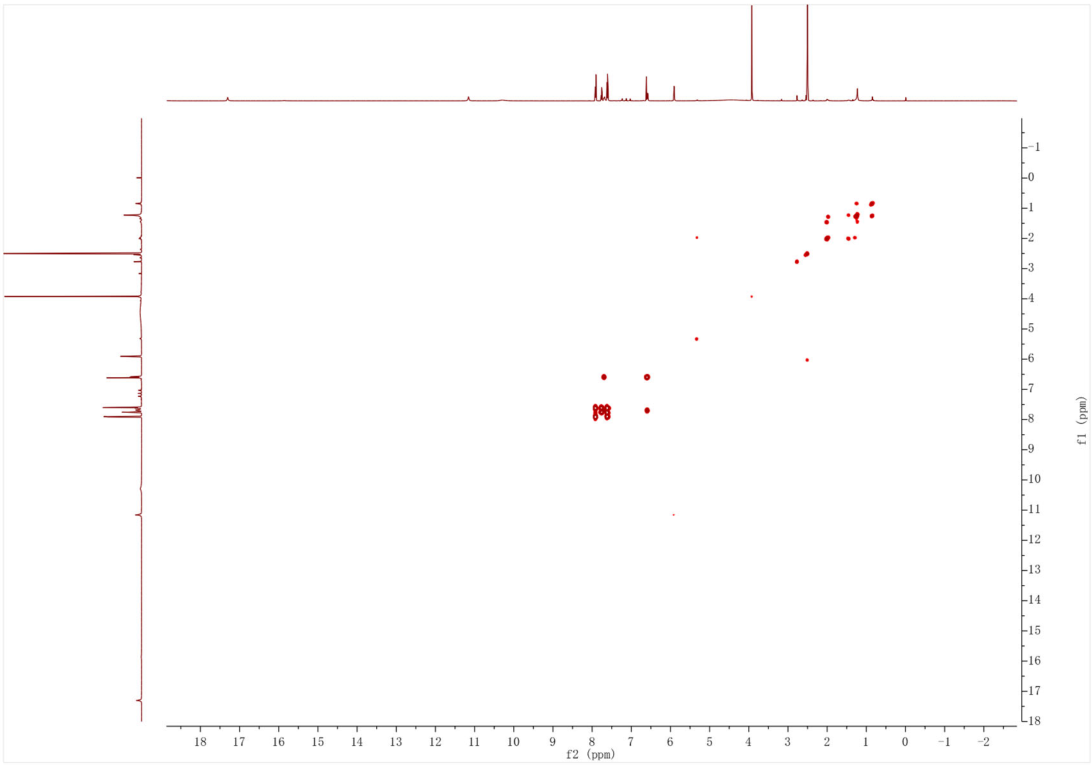

Fig S34: NOESY spectrum of compound 4;

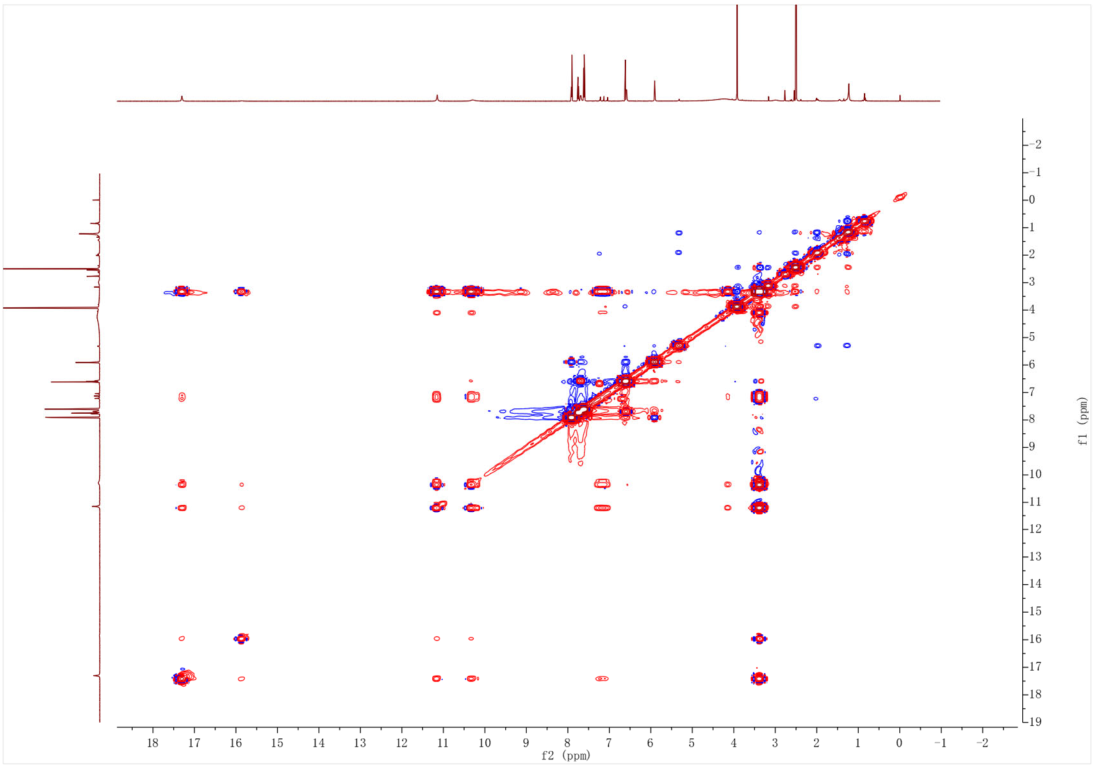

Fig S35: IR spectrum of compound 4;

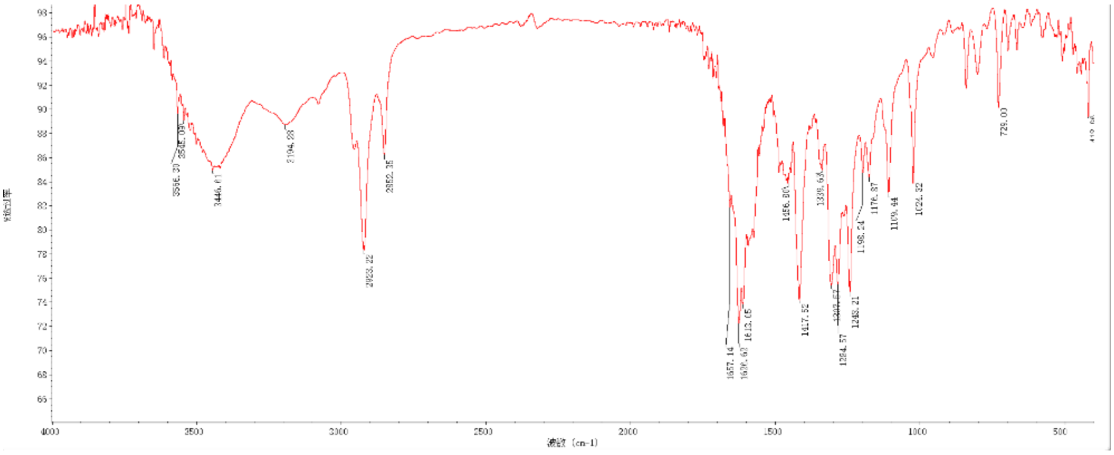

Fig S36: HRESIMS spectrum of compound 5;

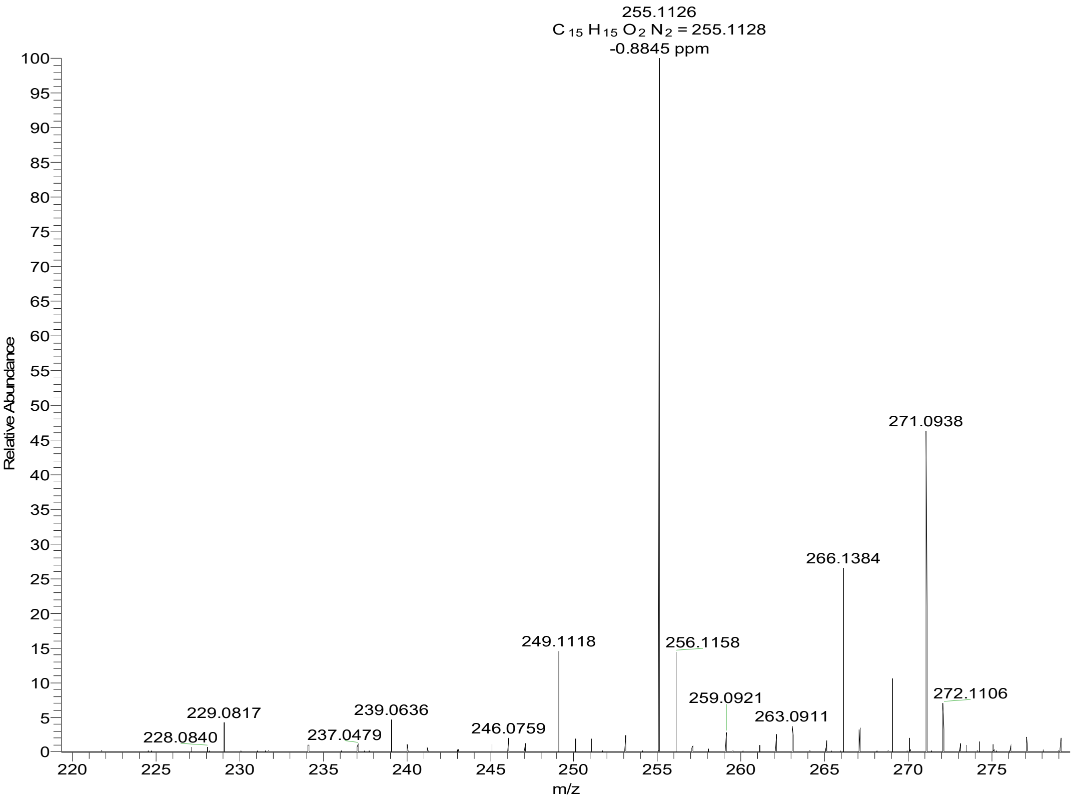

Fig S37: ^{1}H NMR spectrum (400 MHz, CDCl3) of compound 5;

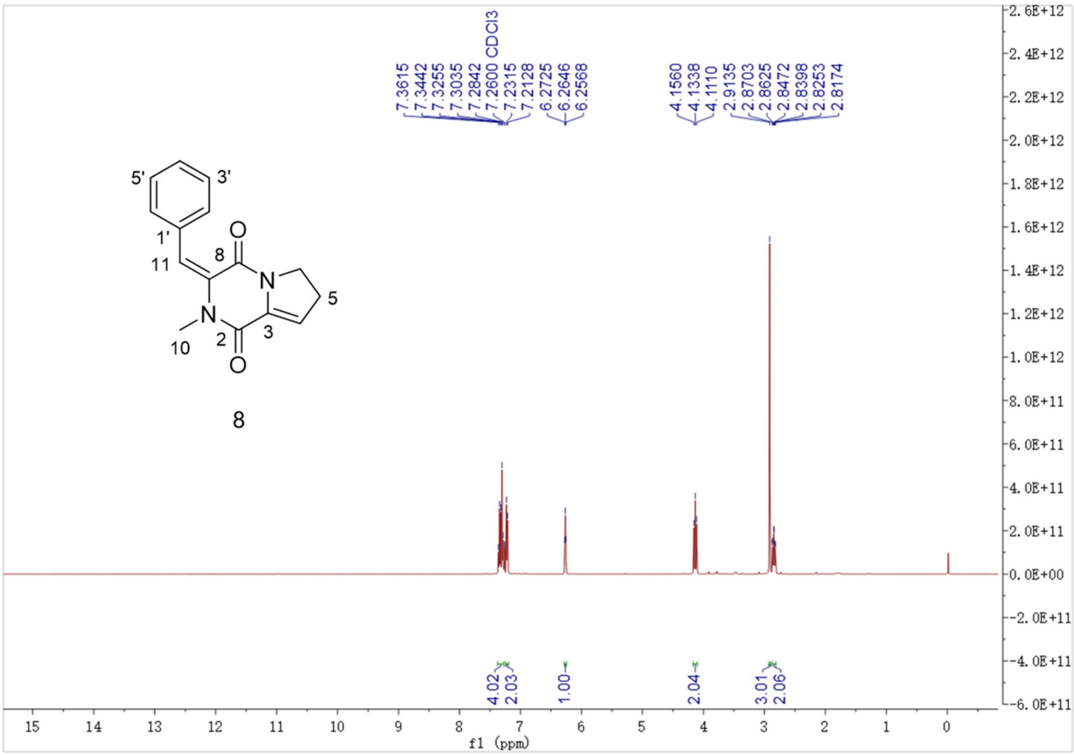

Fig S38: ^{13}C NMR spectrum (100 MHz, $CDCl_3$) of compound 5;

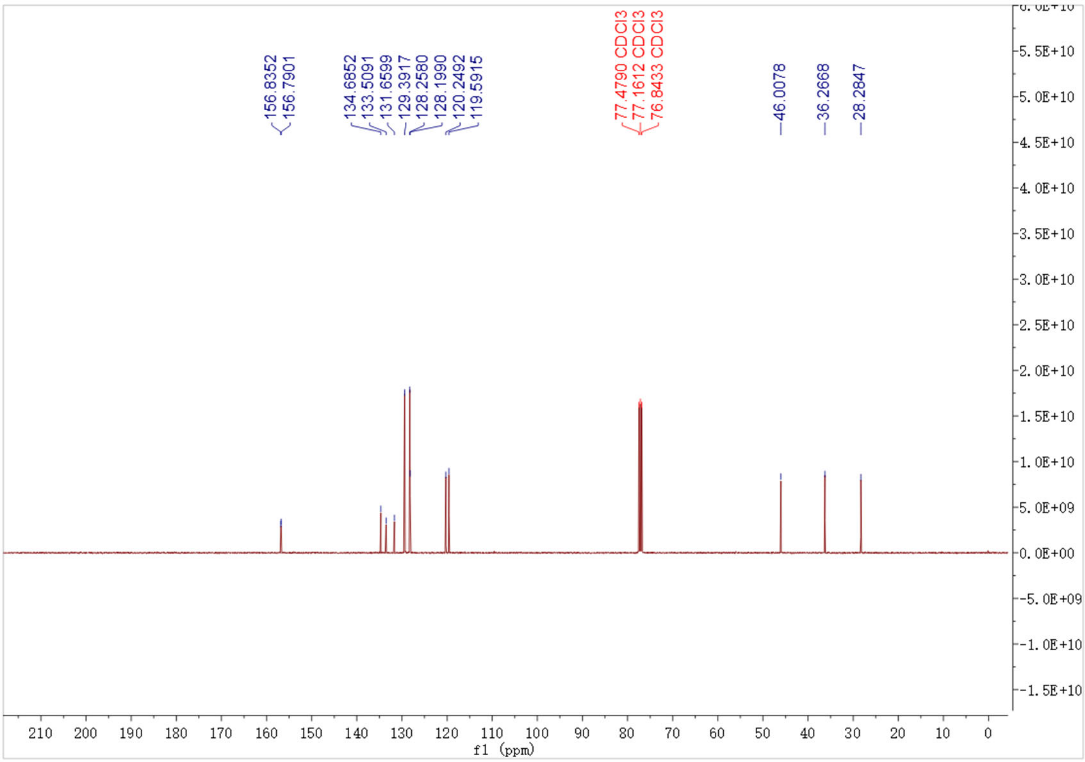

Fig S39: HSQC spectrum of compound 5;

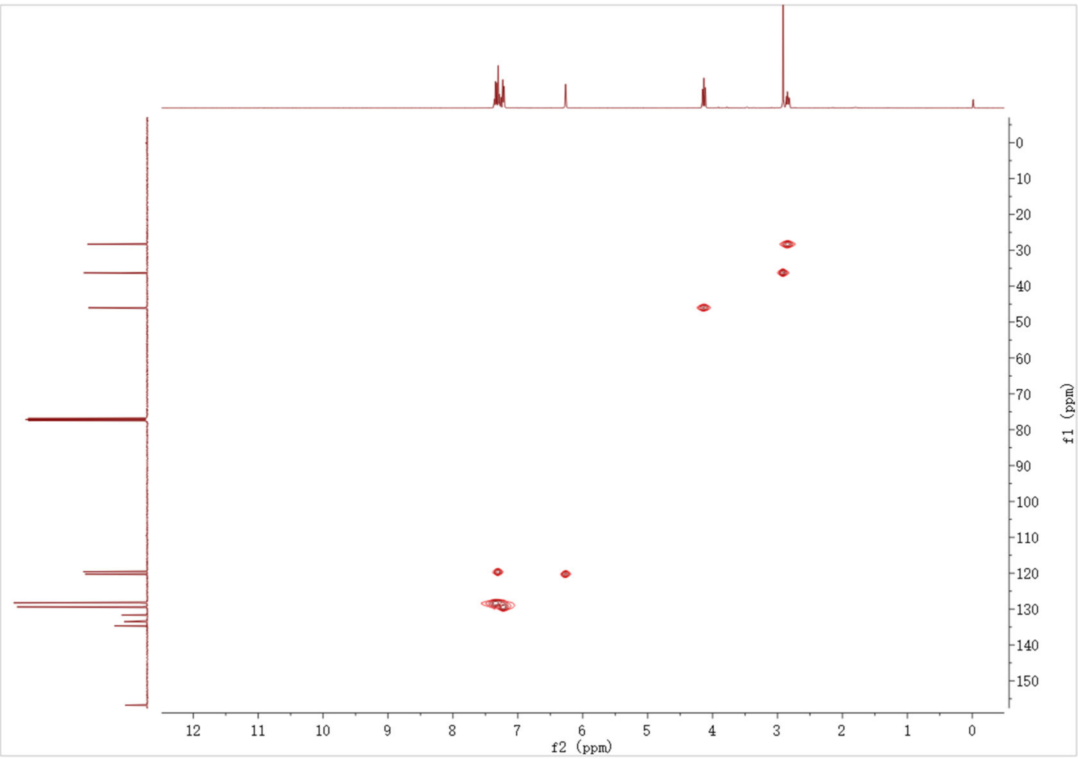

Fig S40: HMBC spectrum of compound 5;

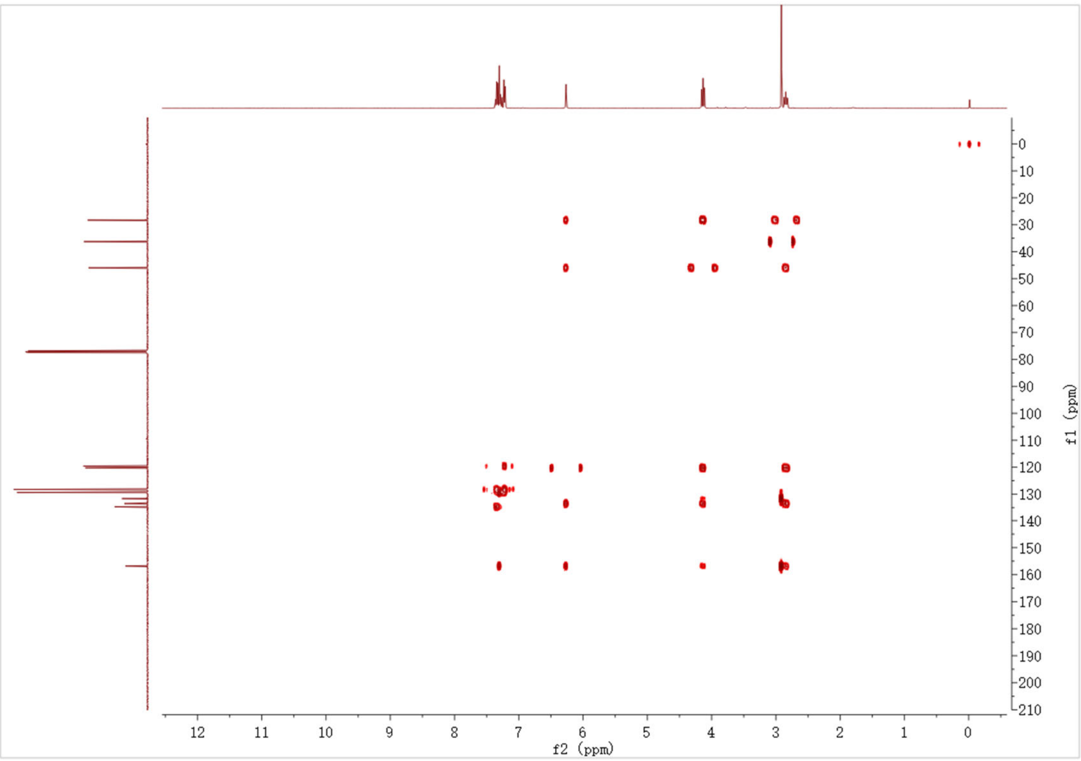

Fig S41: COSY spectrum of compound 5;

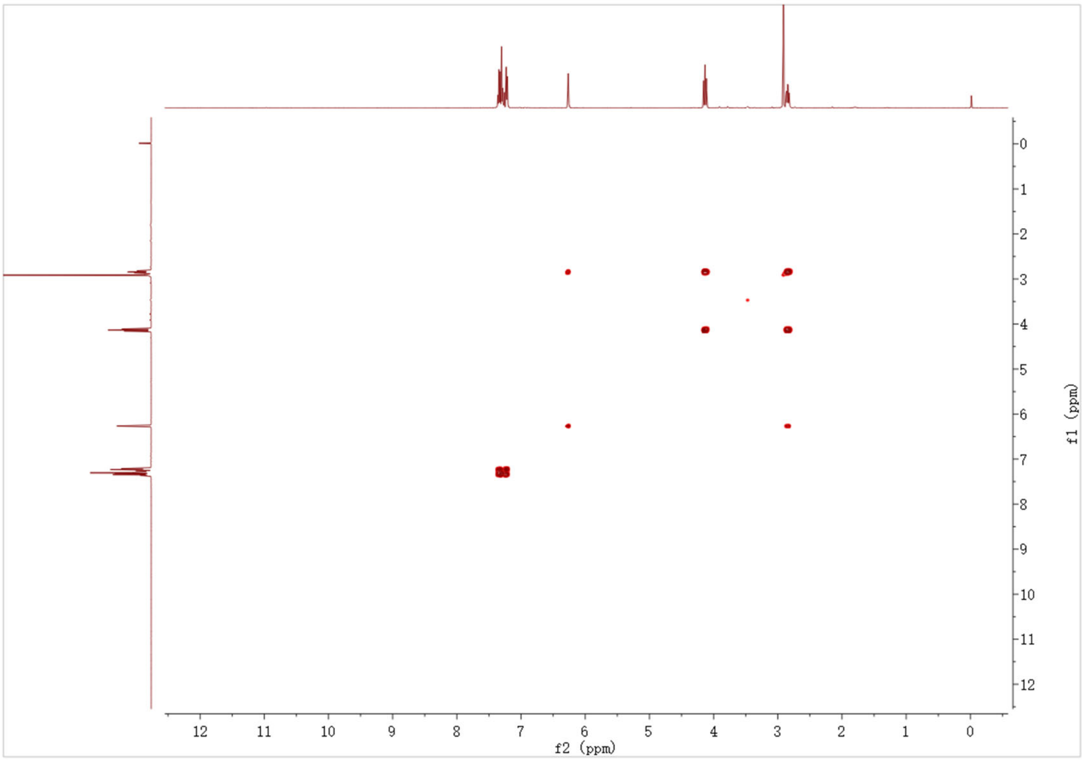

Fig S42: NOESY spectrum of compound 5;

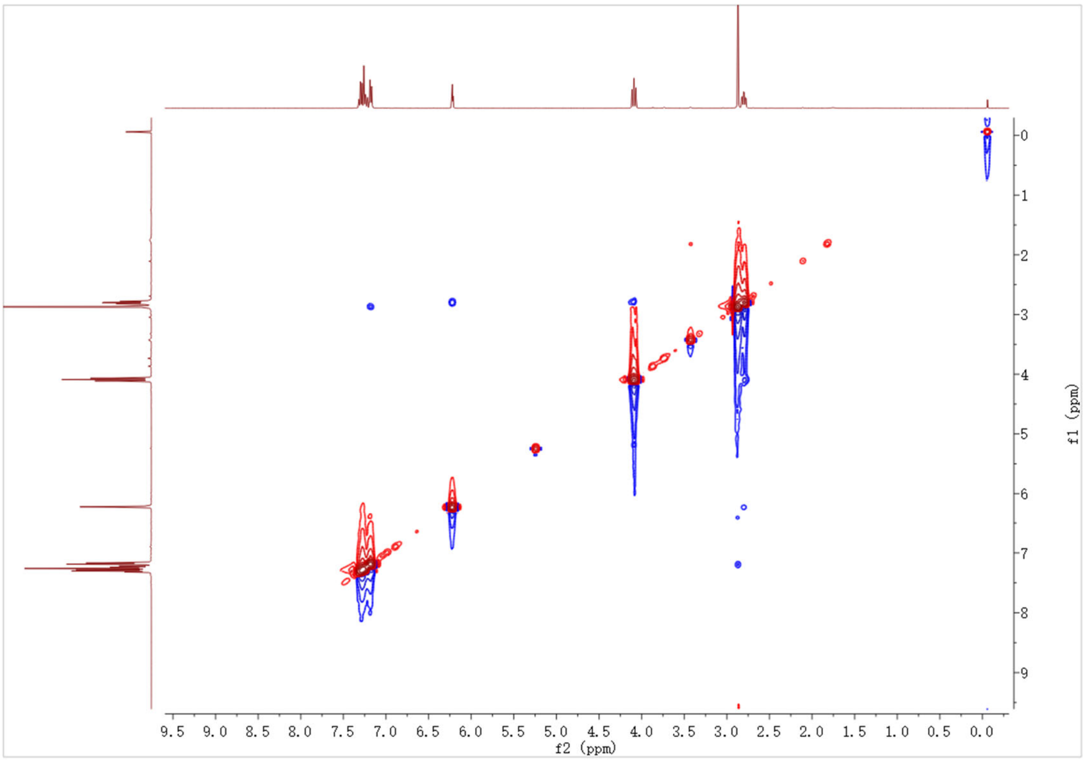

Fig S43: HSQMBC spectrum of compound 5;

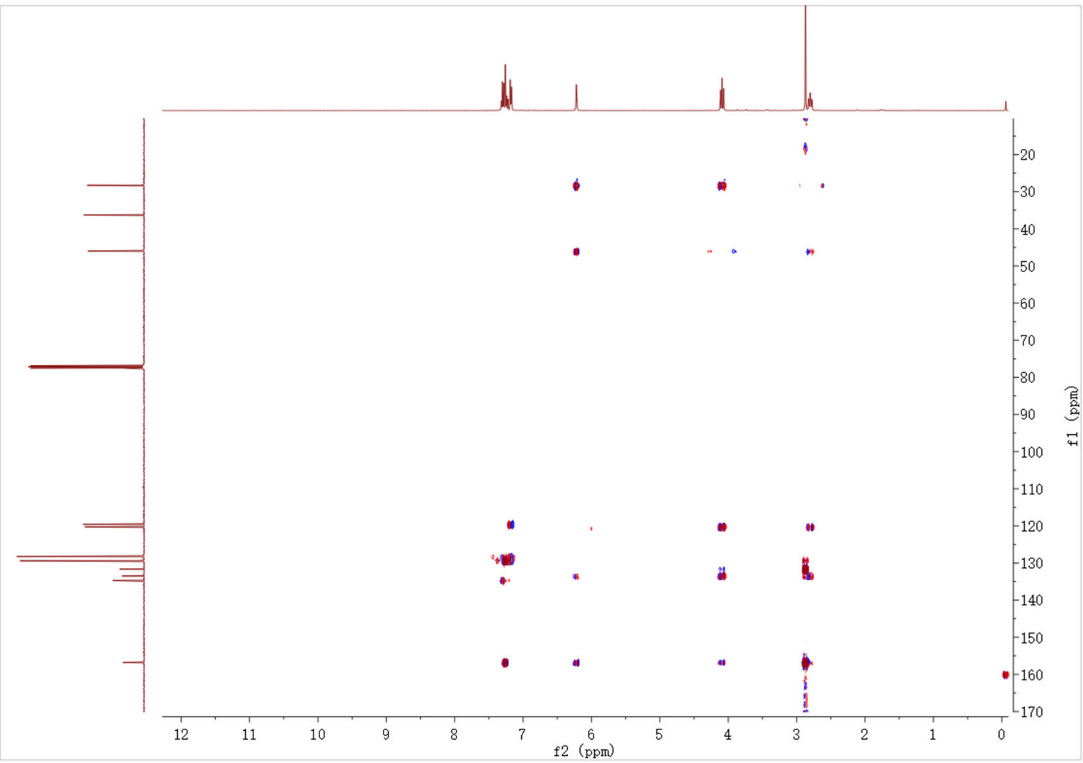

Fig S44: ${}^{3}J_{C\text{-}H}$ values obtained from the HSQMBC spectrum (400 MHz, CDCl₃) of 5.

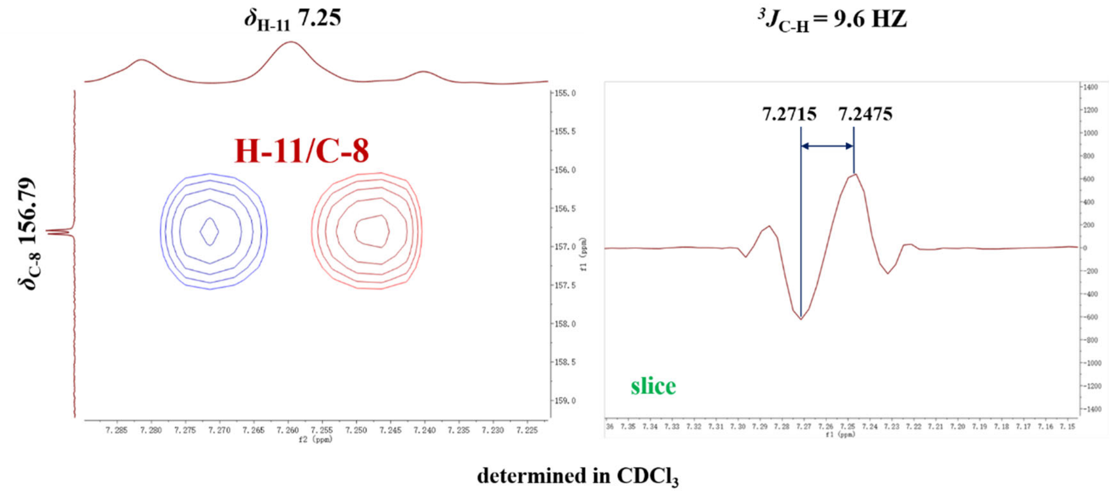

Fig S45: IR spectrum of compound 5;

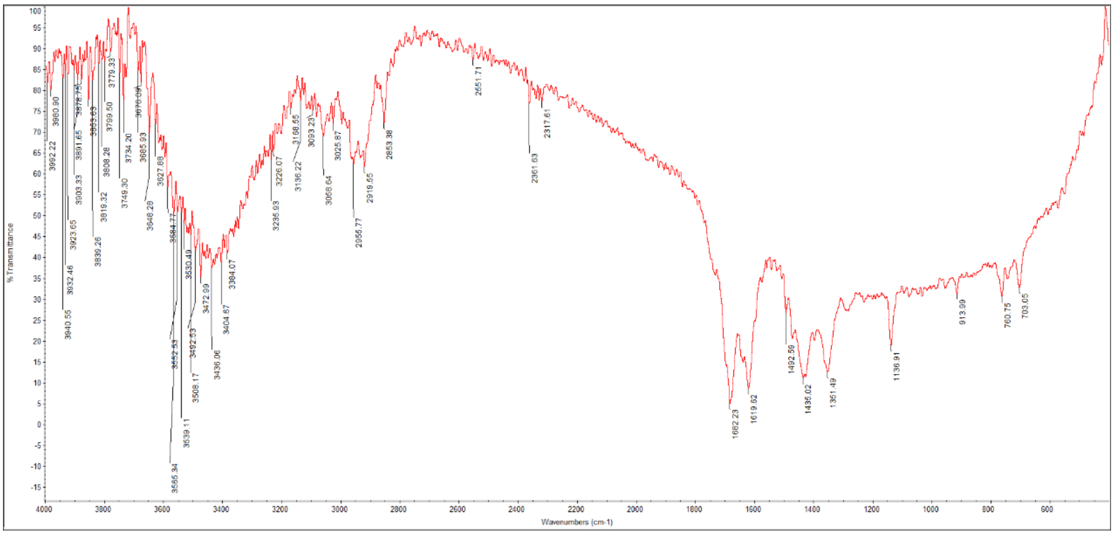

Fig S46: HRESIMS spectrum of compound 6;

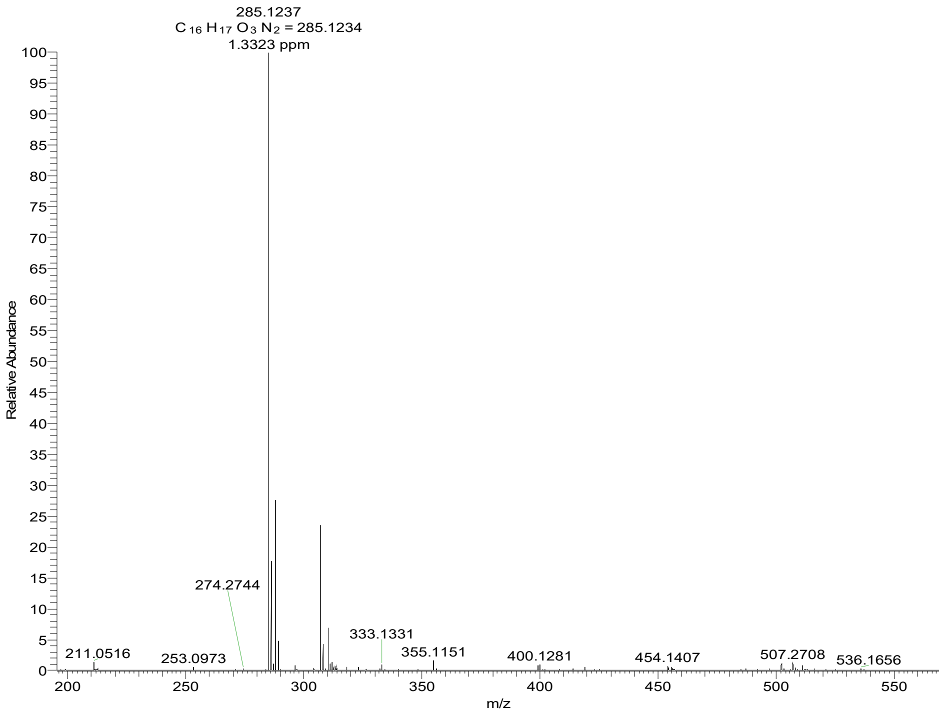

Fig S47: ^{1}H NMR spectrum (100 MHz, CD_3OD) of compound 6;

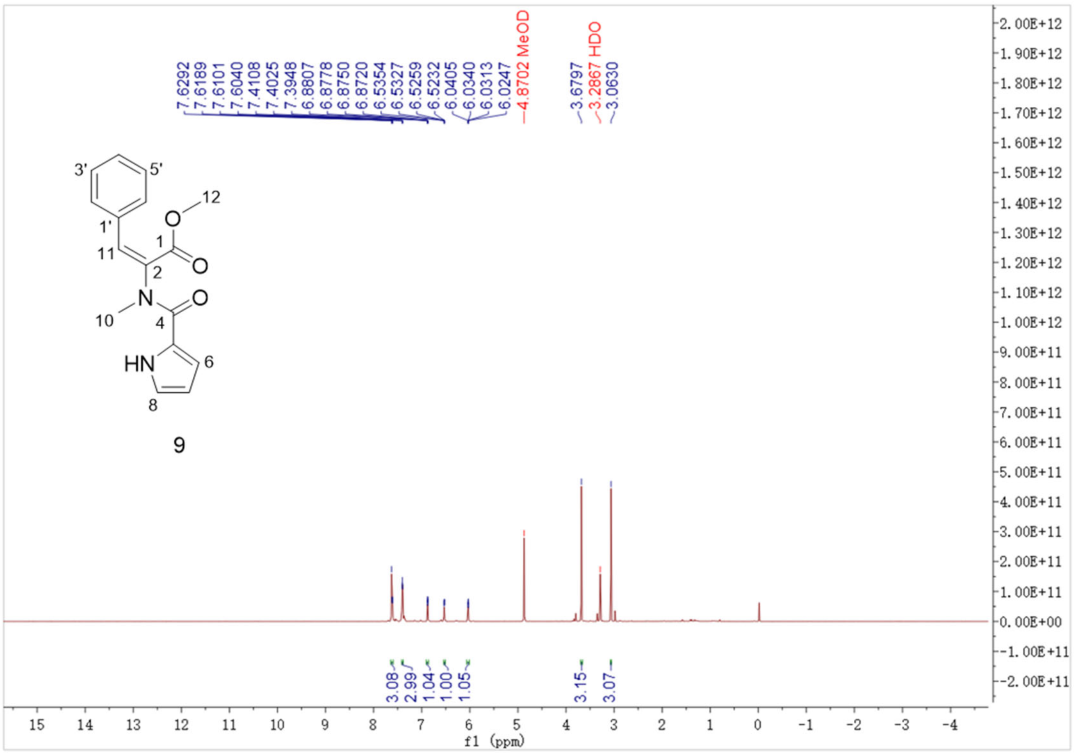

Fig S48: ^{13}C NMR spectrum (100 MHz, CD_3OD) of compound 6;

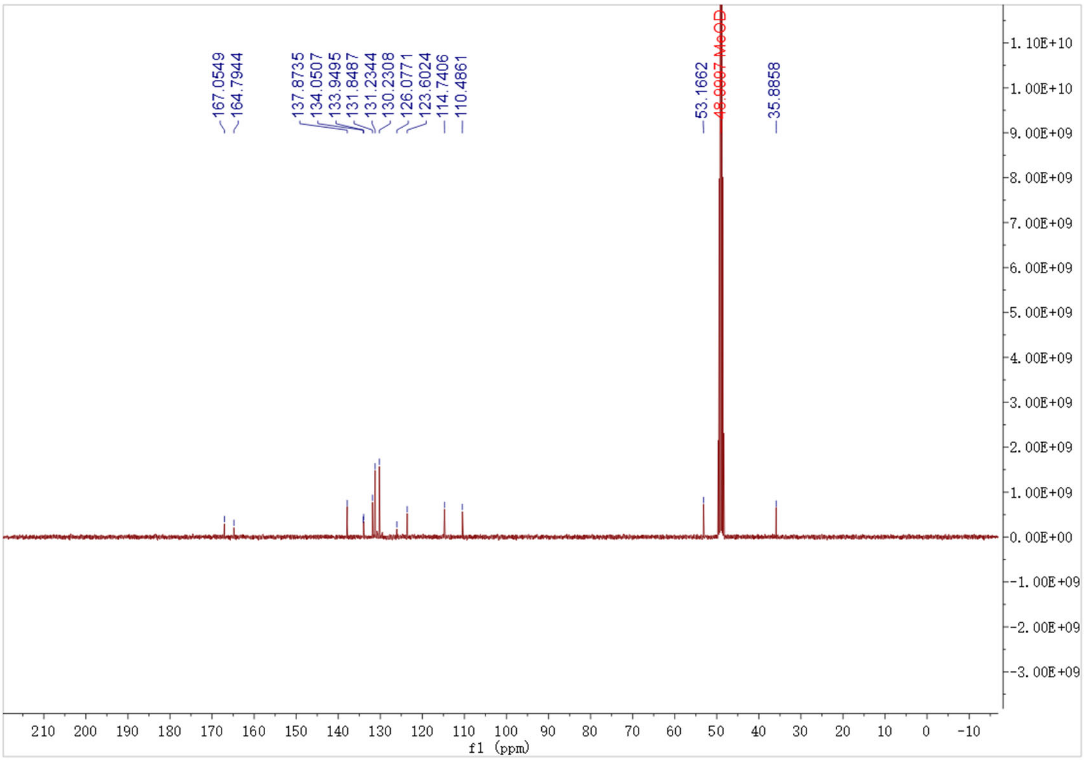

Fig S49: HSQC spectrum of compound 6;

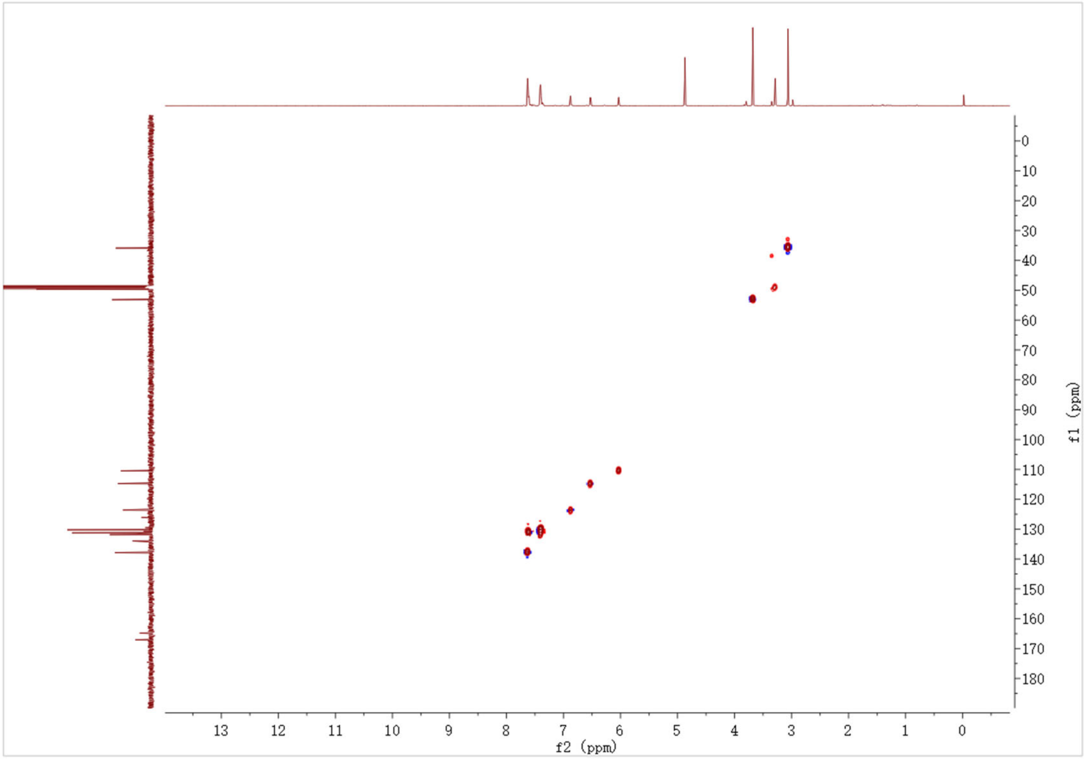

Fig S50: HMBC spectrum of compound 6;

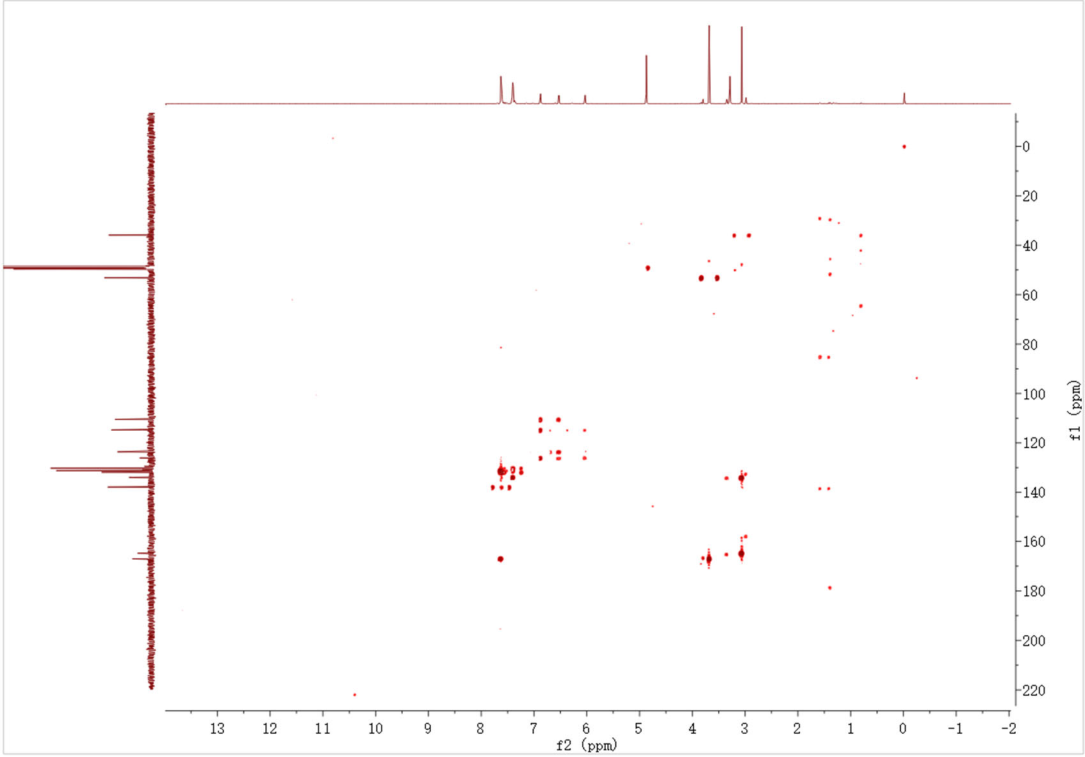

Fig S51: COSY spectrum of compound 6;

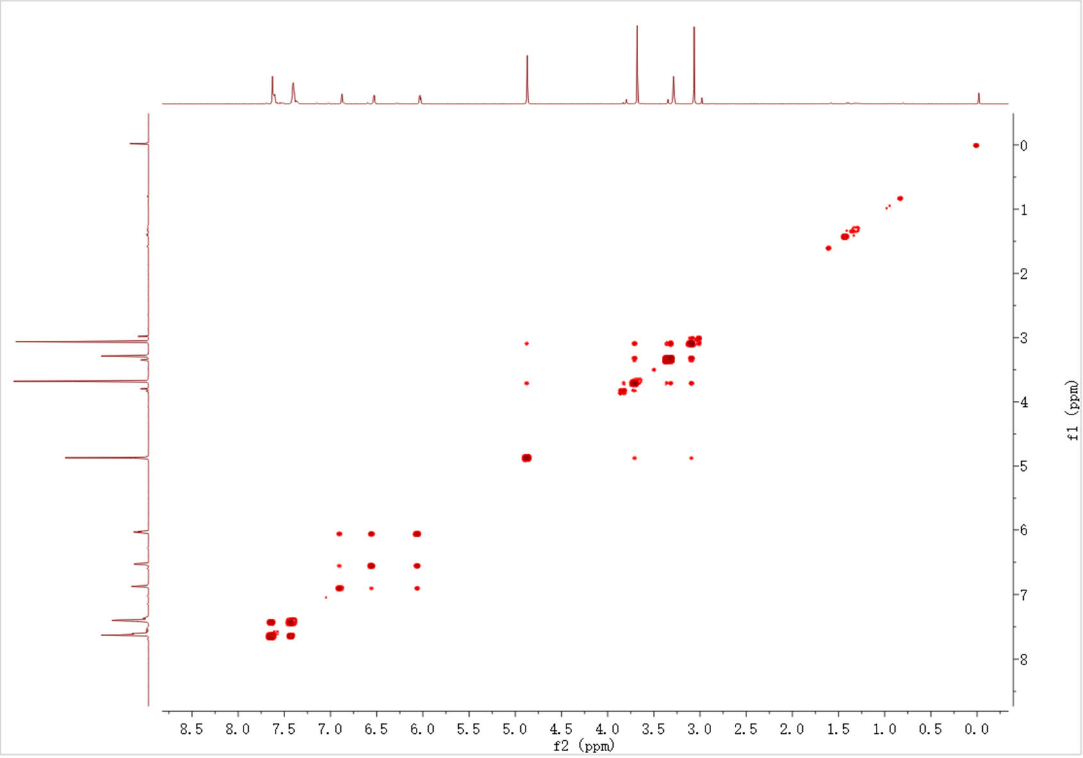

Fig S52: NOESY spectrum of compound 6;

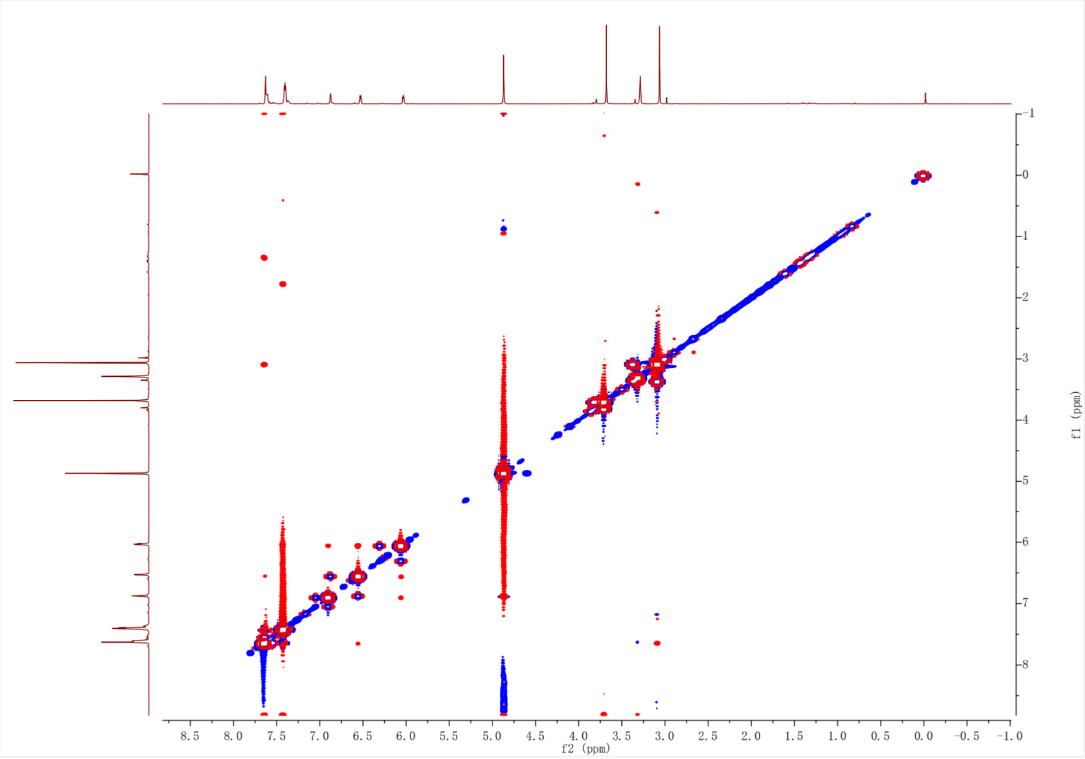

Fig S53: IR spectrum of compound 6;

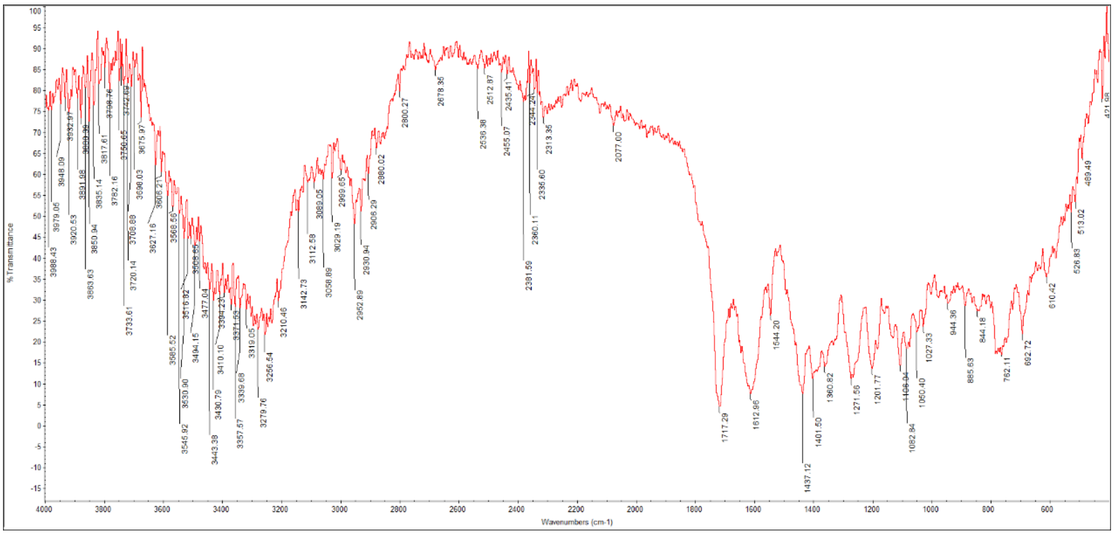

CD Calculation

Conformational analysis was initially performed using SPARTAN'14 software employing the MMFF. All generated conformers were subsequently optimized at the B3LYP/6-31+G(d) level of density functional theory (DFT) using Gaussian 09[1]. The six lowest-energy conformers (each representing >5% of the Boltzmann population) (Figure S54) were then selected for time-dependent DFT (TDDFT) calculations. The ECD spectrum was simulated using the SpecDis program[2]. A Gaussian band shape with a full width at half maximum (FWHM) of 0.3 eV was applied. To facilitate

comparison with experimental data, the calculated spectrum was shifted by +5 nm.

Fig S54: ECD: B3LYP/6-31G(d) optimized lowest energy conformers for 3;

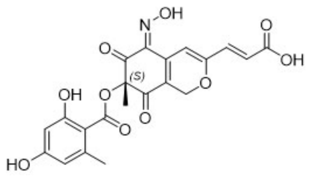

3a

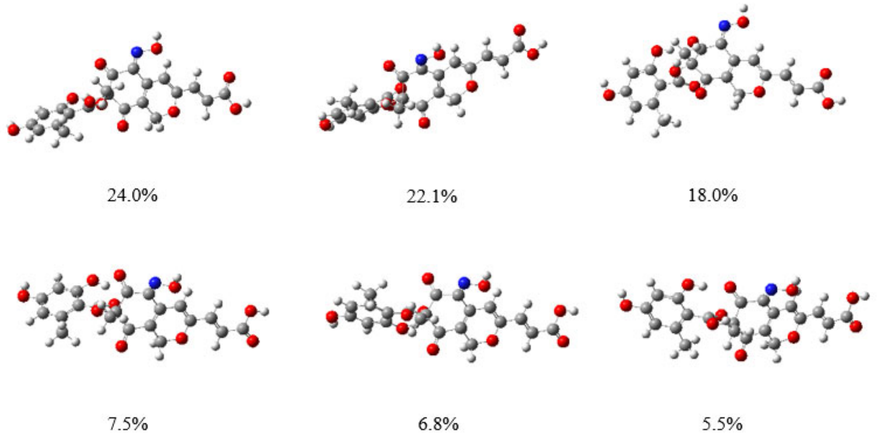

NMR Calculation

The candidate structures of *E*-**3a** and *Z*-**3b** were investigated using quantum chemical DFT calculations to determine their theoretical 1D NMR chemical shifts. Conformational analyses were initially performed through Monte Carlo searching using the molecular mechanism with MMFF force field in the *Spartan 14* program. The results identified six lowest energy conformers for *E*-**3a** and five lowest energy conformers for *Z*-**3b** (Boltzmann Distribution >5%) (Figure S55). These conformers were reoptimized using DFT at the B3LYP/6-31G(d) level in gas phase using the Gaussian 09 program. Gauge-Independent Atomic Orbital (GIAO) calculations of their ^{1}H and ^{13}C NMR chemical shifts were accomplished by Time-dependent DFT (TDDFT) at the B3LYP/6-311G(2d,p) level in PCM (Acetone).

The experimental and calculated data were analyzed using the improved probability DP4+ method (Table S2)[3]. The experimental data of **3** and the calculated data of *E*-**3a** and *Z*-**3b** were analyzed. The final score of *E*-**3a** (0.06%) showed an absolute advantage when comparing with that of *Z*-**3b** (99.94%), indicating that *Z*-**3b** as the correct configuration rather than *E*-**3a** (Table S2).

Fig S55: B3LYP/6-31G(d) optimized lowest energy conformers for 3;

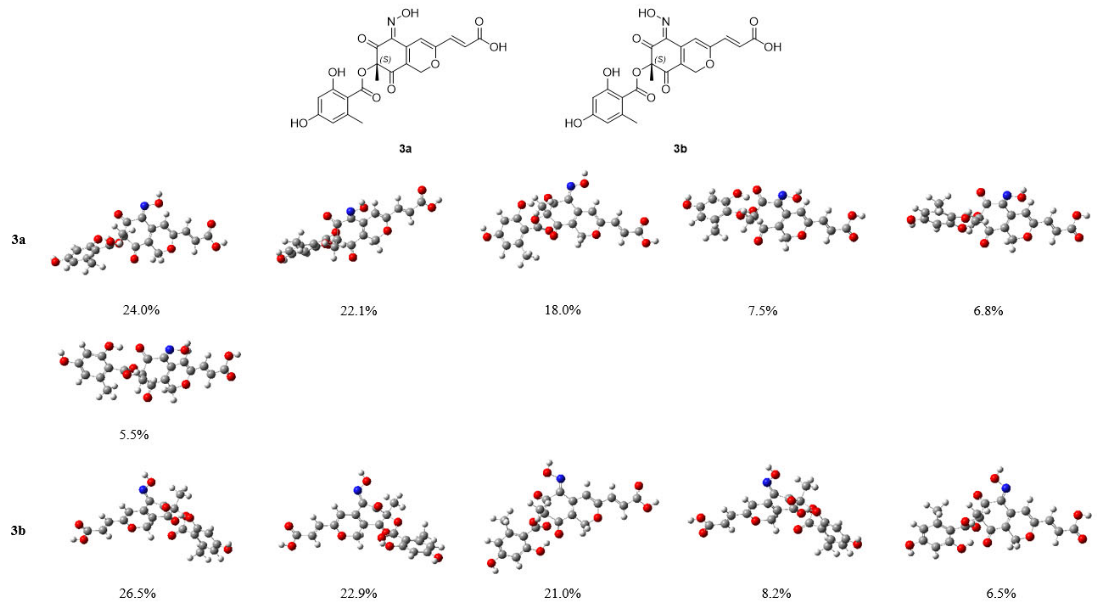

Fig S56: NMR calculations with DP4+ probability analysis for compounds 3.

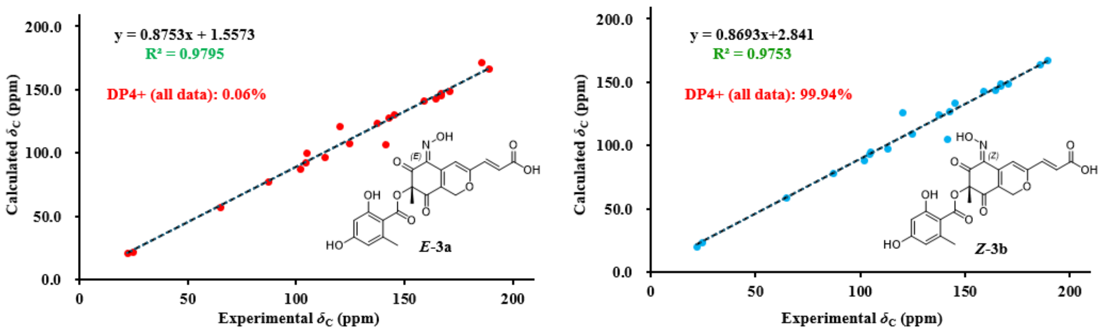

Table S2. DP4+ evaluation of theoretical and experimental one of 3.

			Shielding Tensors	Shielding Tensors
Nuclei	sp2?	Experimental	Isomer 1	Isomer 2
C	x	137.3	58.6006253	57.8284256
C	x	124.4	75.0973046	73.1888206
C	x	166.9	35.1200663	34.8966681
C	x	170.5	33.1872059	33.5103391
C	x	142.4	54.2732008	55.6198569
C	x	104.3	89.7108809	88.524636
C	x	166.6	36.6222347	33.0554195
C	x	101.8	95.0363133	94.3602817
C	x	164.4	39.6096785	38.4056494
C	x	113	85.8280994	84.494582
C		145	52.2390008	48.7058416
C		21.9	161.4081726	161.9547396

C		24.3	160.3196204	158.8563247
C	x	104.9	82.3785927	86.8631083
C	x	158.8	40.6872349	39.1835212
C	x	185.4	11.0203213	18.0647334
C	x	120	61.3371762	55.8579395
C		86.9	104.7779835	103.7613386
C	x	141.3	75.6613641	76.7765043
C	x	189	15.9781524	14.8060896
C		64.6	124.9335779	123.8470694
H	x	6.56	25.6961416	26.1764463
H		5.14	27.7417968	26.8247832
H		5.14	27.0120042	27.827961
H	x	7.23	25.4243495	25.5309731
H	x	6.42	26.121573	26.0485651
H	x	6.25	26.5018198	26.630975
H	x	6.38	26.3110731	26.3638124
H		1.77	30.4927757	30.7292738
H		1.77	30.4500976	30.5336716
H		1.77	30.2255748	30.3128853
H		2.62	30.0005474	30.0917728
H		2.62	29.392421	29.3712517
H		2.62	29.5371692	29.4300152
**Functional**		**Solvent?**		**Basis Set**
**B3LYP**		**PCM**		**6-311+G(d,p)**
		H (sDP4+)	98.46%	1.54%
		C (sDP4+)	0.18%	99.82%
		sDP4+	10.37%	89.63%
		H (uDP4+)	98.05%	1.95%
		C (uDP4+)	0.01%	99.99%
		uDP4+	0.55%	99.45%
		H (DP4+)	99.97%	0.03%
		C (DP4+)	0.00%	100.00%
		DP4+	0.06%	99.94%

The candidate structures of *E*-**4a** and *Z*-**4b** were investigated using quantum chemical DFT calculations to determine their theoretical 1D NMR chemical shifts. Conformational analyses were initially performed through Monte Carlo searching using the molecular mechanism with MMFF force field in the *Spartan 14* program. The results identified four lowest energy conformers for *E*-**4a** and four lowest energy conformers for *Z*-**4b** (Boltzmann Distribution >5%) (Figure S57). These conformers were reoptimized using DFT at the B3LYP/6-31G(d) level in gas phase using the Gaussian 09 program. Gauge-Independent Atomic Orbital (GIAO) calculations of their ^{1}H and ^{13}C NMR chemical shifts were accomplished by Time-dependent DFT (TDDFT) at the B3LYP/6-311G(d,p) level in PCM (DMSO).

The experimental and calculated data were analyzed using the improved probability DP4+ method (Table S3)[3]. The experimental data of **4** and the calculated data of *E*-**4a** and *Z*-**4b** were analyzed. The final score of *E*-**4a** (100.00%) showed an absolute advantage when comparing with that of *Z*-**4b** (0.00%), indicating that *E*-**4a** as the correct configuration rather than *Z*-**4b** (Table S3).

Fig S57: B3LYP/6-31G(d) optimized lowest energy conformers for 4;

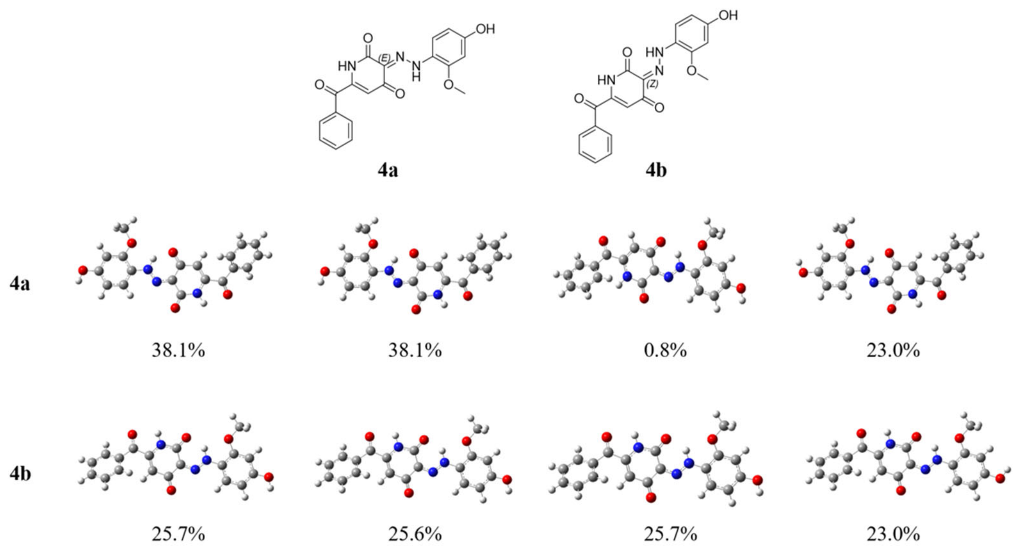

Fig S58: NMR calculations with DP4+ probability analysis for compounds 4.

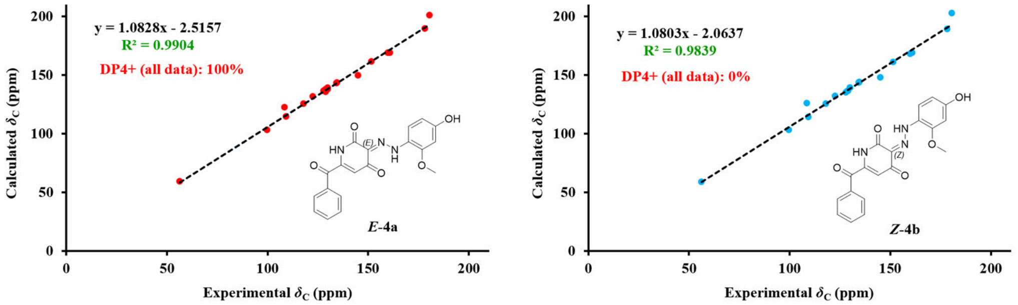

Table S3. DP4+ evaluation of theoretical and experimental one of 4.

			Shielding Tensors	Shielding Tensors
Nuclei	sp2?	Experimental	Isomer 1	Isomer 2
C	x	108.6	61.64255156	58.40004005
C	x	178.2	-5.25950554	-4.82125663
C	x	127.9	47.54351776	48.80720349
C	x	160.9	15.27136252	15.11044929
C	x	122.6	52.66555785	52.08585436
C	x	151.6	22.73172240	23.17575732
C	x	99.7	80.82656114	80.80617390
C	x	159.8	15.43611502	16.07628884

C	x	109.3	69.61063638	69.84903344
C	x	117.9	58.66288767	58.77019421
C		56.3	125.03387007	125.18243020
C	x	180.5	-16.83076304	-18.62337739
C	x	134.6	40.61737848	40.41101784
C	x	129.9	46.57867805	45.29548613
C	x	129	46.57088140	47.67245431
C	x	134.4	41.22953124	40.70938624
C	x	129	48.53103111	47.71329554
C	x	129.9	45.16361332	45.27709406
H		11.15	23.50209135	23.66121546
H	x	5.91	25.48116564	25.54379763
H		17.3	15.85693080	17.08062161
H	x	6.62	25.29031759	25.27000186
H	x	6.59	25.18893480	25.17508729
H	x	7.69	23.66596375	23.74308366
H		10.3	26.66119744	26.74070820
H		3.92	27.42974049	27.44043540
H		3.92	27.92558580	27.93640129
H		3.92	27.92558580	27.93640129
H	x	7.91	23.66451960	23.75993458
H	x	7.61	23.92177935	23.94286195
H	x	7.76	23.81133026	23.76716052
H	x	7.61	24.10054234	23.94532087
H	x	7.91	23.66725521	23.76989375
**Functional**		**Solvent?**		**Basis Set**
**B3LYP**		**PCM**		**6-311+G(d,p)**
		H (sDP4+)	99.89%	0.11%
		C (sDP4+)	99.66%	0.34%
		sDP4+	100.00%	0.00%
		H (uDP4+)	51.82%	48.18%
		C (uDP4+)	99.67%	0.33%
		uDP4+	99.69%	0.31%
		H (DP4+)	99.90%	0.10%
		C (DP4+)	100.00%	0.00%
		DP4+	100.00%	0.00%

References

1. Frisch, M.J.; Trucks, G.; Schlegel, H.B.; Scuseria, G.E.; Robb, M.A.; Cheeseman, J.; Scalmani, G.; Barone, V.; Mennucci, B.; Petersson, G.A.; et al. Gaussian 09 Revision A.1. Gaussian Inc. **2009**.
2. Bruhn, T.; Schaumlöffel, A.; Hemberger, Y.; Bringmann, G. SpecDis: Quantifying the Comparison of Calculated and Experimental Electronic Circular Dichroism Spectra. *Chirality* **2013**, *25*, 243–249.
3. Grimblat, N.; Zanardi, M.M.; Sarotti, A.M. Beyond DP4: An Improved Probability for the Stereochemical Assignment of Isomeric Compounds Using Quantum Chemical Calculations of NMR Shifts. *J. Org. Chem.* **2015**, *80*, 12526–12534.
